# Comparison of key quality characteristics and table grape potentials of diploid and triploid hybrid genotypes obtained through cross-breeding

**DOI:** 10.1186/s12870-026-09389-7

**Published:** 2026-07-24

**Authors:** Selin Ateş, Onur Ergönül, Aslı Polat, Gamze Uysal Seçki̇n, Eküle Sönmez, Aydın Uzun, Arif Atak

**Affiliations:** 1 Graduate School of Natural and Applied Sciences, Bursa Uludağ University, Bursa, 16059 Turkey; 2 Tekirdağ Viticulture Research Institute, Tekirdağ, 59000 Türkiye; 3 Department of Horticulture, Faculty of Agriculture, Bursa Uludağ University, Bursa, 16059 Türkiye; 4 Department of Horticulture, Faculty of Agriculture, Erciyes University, Kayseri, 38030 Türkiye; 5 Projenia R&D and Consulting Co. Ltd, Erciyes Technopark, Kayseri, 38010 Türkiye

**Keywords:** Seedlessness, Polyploidy, Berry size, Quality, Sensory test

## Abstract

In many countries worldwide, new grape varieties are being developed through various breeding programs to meet changing consumer demands in the table grape market. In Türkiye, one of the countries with the most widespread table grape production, breeding studies are ongoing in various institutions. These studies generally involve hybridization within *V.vinifera* and between different species. These hybridizations result in hybrid genotypes with different characteristics. In this study, 51 intraspecific hybrid genotypes and 6 interspecific triploid hybrid genotypes developed through hybridization breeding at the Tekirdağ Viticulture Research Institute were compared with 6 standard varieties on 30 quality characteristics. Data were obtained from hybrid genotypes grafted onto 1103 Paulsen rootstocks, in the second stage, and possessing a sufficient number of clusters. Among these characteristics, seedlessness, large size, unique aroma, degustation test (sensory score), and harvest time have been the most prominent selection criteria. The aim of the research is to identify potential table grape varieties with market value and the potential to generate high income for table grape growers. Among the hybrid genotypes evaluated, 44XD-8 stood out particularly for its large berry size, herbaceous aroma, strong berry-stem connection, early maturity, and disease resistance. 44XT-26, on the other hand, stood out with its large, seedless berries, early maturity, and high sensory test score. Additionally, 7XM-98 was another hybrid genotype that stood out for its high sensory test scores and late maturity. KPXAK-26, 41XT-25, 41XGG-6, 7XB-60, 43XB-103, 43XB-119, and TSXBARIŞ-31, which scored 15 or higher in sensory tests compared to standard varieties such as Tekirdağ Çekirdeksizi and Trakya İlkeren, were other promising hybrid genotypes. Based on the results of advanced quality analyses of these promising hybrid genotypes, they could be registered and transferred to grape growers in the near future.

**Supplementary Information:**

The online version contains supplementary material available at 10.1186/s12870-026-09389-7.

## Introduction

According to the current FAO database, vineyards worldwide cover an area of approximately 6.7 million hectares, producing around 76 million tons. Türkiye has a significant share of approximately 5% in this production [1]. Despite these production figures, Türkiye is far behind the desired levels in table grape exports. The most important reasons for this situation include the fact that the Sultani seedless variety, which dominates production, is largely used for raisin and is not of a quality that can compete with newly developed table grape varieties [2, 3]. To meet rapidly changing consumer and market demands in the table grape market, various breeding programs are being implemented. The main objectives of these programs include developing varieties with larger berries, seedlessness, firmer flesh, different tastes, and greater tolerance to various diseases [4]. Cross-breeding is the process of mating two different grape varieties, of the same or different species, to produce offspring (hybrids). Its primary purpose is to combine the best traits of both parent lineages to create stronger, healthier, or more productive hybrids—a phenomenon known as hybrid vigor [5].

Developing new grape varieties is the most efficient way to meet market expectations, compared to identifying mutated varieties among existing ones. Different breeding programs are being conducted to improve table grapes in various ways and select new varieties with superior characteristics. Countries like Spain and the USA, in particular, have developed quite high-quality new table grape varieties since the last century [6]. In parallel with changes in consumption habits, the demand for new, superior table grape varieties that can meet these expectations is increasing [7]. In recent years, new table grape varieties developed through breeding studies conducted by state and public institutions have begun to be commercially produced in many countries and have yielded high profits [8]. The main diseases causing the most economic damage to grapevines are powdery mildew and downy mildew. Many different spraying applications are used to combat them, which increases costs and sometimes fails to achieve the desired results. In recent years, developing new table grape varieties resistant to downy and powdery mildew has become one of the most important breeding goals in table grape breeding programs [9, 10]. Cultivated grapevines are generally susceptible to these pathogens, and sources of resistance come from wild species in America and Asia [5, 11].

Through polyploidization studies, it is possible to improve the quality and yield of grapes by increasing the chromosome number using different methods in both seeded and seedless varieties [12, 13]. Furthermore, it has become a frequently preferred breeding method in recent years because it provides genetic improvement in many plant species, including grapes, without significant loss of superior traits, and allows rapid achievement of desired results, as well as resistance to biotic/abiotic stress conditions in breeding programs [14, 15].

Grape breeding studies in Türkiye began in 1973 and continue today in various institutions and organizations, primarily focusing on table grape breeding. Almost every year, new grape varieties are registered and made available to grape growers [16]. The key quality characteristics of newly developed hybrid grapes should be investigated to determine their suitability for consumer and market expectations. In particular, examining berry and Cluster characteristics, conducting sensory tests to identify outstanding varieties, and prioritizing these varieties in registration efforts are crucial to aligning with consumer preferences [17, 18].

In this study, the cluster and berry characteristics of 57 hybrid grape genotypes in the second stage of breeding studies conducted at the Tekirdağ Viticulture Research Institute, which has been working on table grape breeding in Türkiye for many years, and 6 triploid genotypes developed from polyploidy breeding studies were examined, and comparisons were made with 6 different standard varieties.

## Material and method

### Material

This study was conducted in the second stage plot (40°97’24.12” North and 27°47°21.22” East) of the experimental vineyard belonging to the Tekirdağ Viticulture Research Institute.

This study mainly involves hybridization(cross-breeding) within *V.vinifera* and between different species. As a result of these cross-breedings, hybrid grape genotypes belonging to the *V.vinifera* species with different characteristics were obtained. In this study, 51 intraspecific hybrid genotypes and 6 interspecific triploid hybrid genotypes developed through hybridization breeding at the Tekirdağ Viticulture Research Institute were compared with 6 standard varieties on 30 quality characteristics. Data were obtained from hybrid genotypes grafted onto 1103 Paulsen rootstocks, in the second stage, and possessing a sufficient number of clusters.

The rightful owner of all plant materials is the Tekirdağ Viticulture Research Institute. The material for this study consists of 51 hybrid grape genotypes aged 9 years, crossed with 22 different combinations, and 6 triploid hybrids aged 6 years. 43 of the hybrid genotypes were seedless, and only 14 were seeded (Table 1). The 9-year-old hybrid grapevines are grafted onto 1103 P rootstock in the second-stage plot at the Institute, with 3–5 vines of each variety candidate. The 6-year-old triploid hybrids are on their own roots in 30-liter pots. In this study, a total of six reference varieties were used for grapes ripening in three different periods: early, mid, and late. These reference varieties were: one seeded variety and one seedless variety.

**Table 1:** Combination numbers of hybrid genotypes and their parents used in the study

No	Combination Code	Parents (Combinations)
1	44XT-26	Yalova İncisi X Tekirdağ Çekirdeksizi
2	ASXM-5	Atasarısı X Reçel Üzümü
3	240XR-6	240 X Tekirdağ Misketi
4	37XT-163	Çınarlı Karası X Tekirdağ Çekirdeksizi
5	44XD-8^*^ (Günışığı)	Yalova İncisi X Barış
6	44XD-25	Yalova İncisi X Barış
7	YİXTM-3	Yalova İncisi X Tekirdağ Misketi
8	44XD-40	Yalova İncisi X Barış
9	7XD-258	Italia X Barış
10	37XD-25	Çınarlı Karası X Barış
11	7XM-98^*^	Italia X Reçel Üzümü
12	44XD-31^*^	Yalova İncisi X Barış
13	7XB-60	Italia X Perlette
14	7XB-79	Italia X Perlette
15	40XD-160	İrikara X Barış
16	41XM-10	Red Globe X Reçel Üzümü
17	TSXBARIŞ-31	Tekirdağ Sultanı X Barış
18	7XC-137	Italia X Beauty Seedless
19	44XD-12	Yalova İncisi X Barış
20	43XB-119	Hönüsü X Perlette
21	43XB-103	Hönüsü X Perlette
22	43XB-84^*^	Hönüsü X Perlette
23	43XB-42	Hönüsü X Perlette
24	40XD-91	İrikara X Barış
25	40XD-17	İrikara X Barış
26	40XB-99	İrikara X Perlette
27	41XGG-1	Red Globe X Güz Gülü
28	41XGG-6^*^	Red Globe X Güz Gülü
29	43XB-41	Hönüsü X Perlette
30	40XD-85^*^	İrikara X Barış
31	37XM-8	Çınarlı Karası X Reçel Üzümü
32	ALFXM-3	Alphonse Lavallée X Reçel Üzümü
33	TİXBARIŞ-3	Trakya İlkeren X Barış
34	41XGG-3	Red Globe X Güz Gülü
35	41XT-20	Red Globe X Tekirdağ Çekirdeksizi
36	41XT-25	Red Globe X Tekirdağ Çekirdeksizi
37	41XT-9^*^	Red Globe X Tekirdağ Çekirdeksizi
38	46XM-3	Bozbey X Reçel Üzümü
39	46XL-8^*^	Bozbey X Güz Üzümü
40	46XM-4	Bozbey X Reçel Üzümü
41	46XT-10^*^	Bozbey X Tekirdağ Çekirdeksizi
42	46XT-2	Bozbey X Tekirdağ Çekirdeksizi
43	KPXAK-21^*^	Kadın Parmağı X Antep Karası
44	KPXAK-26^*^	Kadın Parmağı X Antep Karası
45	KPXAK-103^*^	Kadın Parmağı X Antep Karası
44	KPXAK-109^*^	Kadın Parmağı X Antep Karası
45	EXSS-62^*^	Emirali X Superior Seedless
46	KY X Yİ	Kyoho X Yalova İncisi
47	EXT-37	Emirali X Tekirdağ Çekirdeksizi
48	EXT-33	Emirali X Tekirdağ Çekirdeksizi
49	36XL-95	Ribol X Güz Üzümü
50	4^**^	Trakya İlkeren X Kyoho (4n)
51	9^**^	Trakya İlkeren X Kyoho (4n)
52	12^**^	Trakya İlkeren X Kyoho (4n)
53	14^**^	Trakya İlkeren X Kyoho (4n)
54	18^**^	Trakya İlkeren X Kyoho (4n)
55	81^**^	Trakya İlkeren X Kyoho (4n)
56	Red Globe^*^	Reference (late season variety)
57	Kyoho (4n) ^*^	Reference (mid season variety)
58	Crimson Seedless	Reference (late season seedlessvariety)
59	Trakya İlkeren^*^	Reference (early season variety)
60	Tekirdağ Çekirdeksizi	Reference (mid season seedless variety)
61	Alphonse Lavallée^*^	Reference (late season variety)
62	Superior Seedless	Reference (early season seedless variety)

*seeded hybrid genotype/variety, **triploid hybrid genotype

**Fig. 1:**
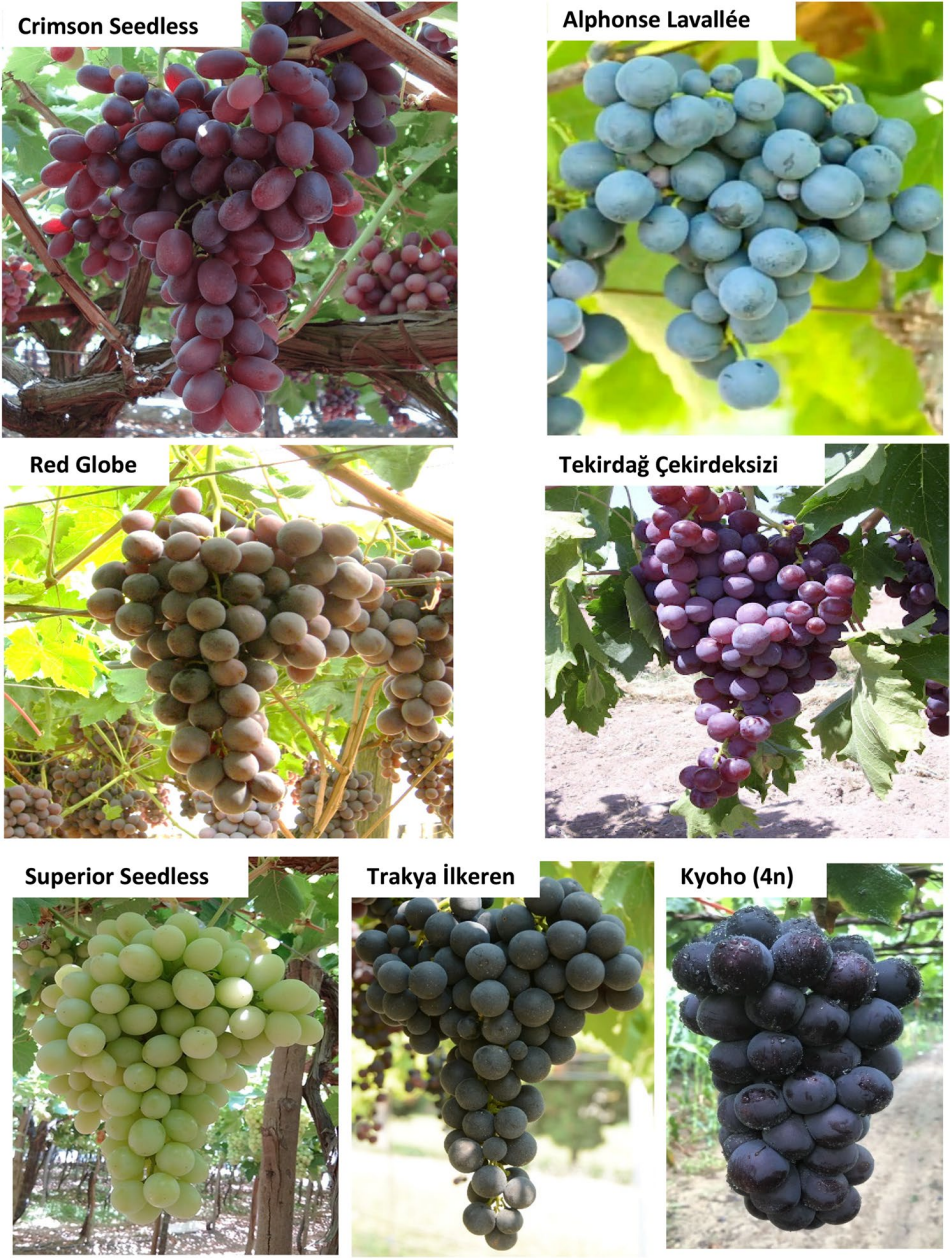
Photos of grape clusters from reference grape varieties used in comparing hybrids

Seven reference varieties were used: ‘Trakya İlkeren’ and ‘Superior Seedless’ for early ripening varieties, ‘Alphonse Lavallée’, ‘Kyoho(4n)’ and ‘Tekirdağ Çekirdeksizi’ for mid-season ripening, ‘Red Globe’ and ‘Crimson Seedless’ for late ripening (Fig. 1).

The varieties and genotypes listed in Table 1 were used as parents in the study due to their different superior characteristics. The main reasons for selecting the parent varieties used in the study are as follows: Yalova İncisi: early ripening; Tekirdağ Seedless: large berry and seedless; Atasarısı: Very large berry and productive; Reçel Üzümü: Seedless and productive; Tekirdağ Misketi: Seedless, aromatic, and medium early; Çınarlı Karası: Late and suitable for long storage; Barış: Seedless and productive; Italia: Large berry and Muscat aroma; Perlette: Early seedless; Tekirdağ Sultanı: Muscat aroma; Hönüsü: Female flower and large berry; İrikara: Large berry, suitable for long storage and late; Güzgülü: Seedless and late; Alphonse Lavallée: Large berry, suitable for storage and high yield; Trakya İlkeren: Very early; Bozbey: Large berry; Kadın parmağı: Large and long berry; Antep karası: Large and long berry; Emirali: Very late and large berry; Ribol: Large berry; Kyoho(4n): Tetraploid, large berry, disease tolerance.

## Method

### Climatic conditions of the experimental site

The experimental site is located in Tekirdağ province. Tekirdağ province has hot summers and mild and rainy winters. Meteorological data during the harvest period of the study were obtained from the nearest meteorological station and are given in Table 2.

**Table 2:** Meteorological data of the experimental site during the vegetation period

Location / Month	Temperature (°C)			Relative Humidity (%)			Rain (mm)	Wind Speed (m/s)		
Tekirdağ VRI	Avg.	Max.	Min.	Avg.	Max.	Min.	Total	Avg.	Min.	Max.
April 2025	10.50	23.34	-2.66	78.07	99.29	17.13	47.4	1.27	7.80	14.90
May 2025	16.69	30.04	3.27	69.71	99.34	20.79	30.8	1.15	5.70	11.10
June 2025	23.33	35.08	11.72	53.08	99.60	21.02	0.2	0.90	3.30	9.20
July 2025	25.85	38.5	12.01	56.52	99.24	16.38	0.0	1.01	4.60	10.60
August 2025	24.99	34.71	14.15	61.63	99.26	14.92	13.4	1.12	4.60	10.10
September 2025	20.79	33.03	10.03	67.01	99.23	24.27	10.4	0.90	3.60	9.90

### Pomological evaluations of clusters

In this study, the characteristics of cluster, berry, and seed structures of hybrid genotypes were examined. Evaluations were made using the scales in the “Second Edition of the OIV Descriptor List for Grape Varieties and *Vitis* Species, 2001 [19]. The scale values and references of the measurements are indicated in the Tables.

#### Cluster weight

Determined in grams by weighing all three randomly harvested clusters and calculating the average Cluster weight.

#### Number of berries per cluster

The number of berries on each of the three randomly harvested clusters was counted, and the number of berries per cluster was determined.

#### Cluster density

**Fig. 2:**
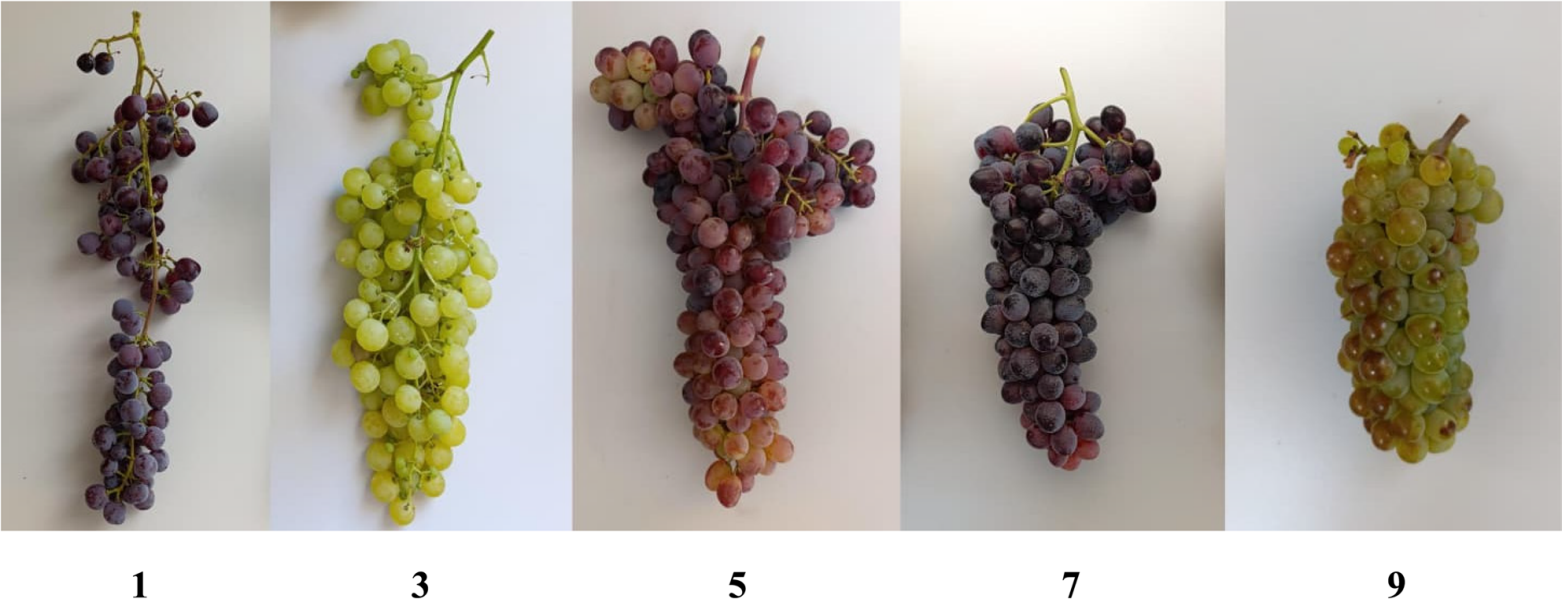
Cluster density scale values and corresponding cluster images

Observation of cluster density according to the OIV 204 scale on randomly harvested clusters during the harvest period. Classification was made according to the scale in Table 3; Fig. 2.

**Table 3:** Cluster density identification criteria and scale values (OIV 204)

Very Loose (berries are distinctly separate, most of the stalks are visible)	1
Loose (berries are lightly touching each other, some of the stalks are visible)	3
Medium (berries are close enough to move, stalks are not visible)	5
Dense (berries are too dense to move)	7
Very Dense (berry shape is distorted due to density)	9

### Analysis of berry structure

#### Soluble solids concentration (°Brix, as SSC)

The total soluble solids content in grape berries was measured using an Atago 0–32 °Brix temperature compensating refractometer (Atago Co., Tokyo, Japan). Samples were selected from the harvested berries, crushed, and the resulting must was filtered. After filtering, the TSS was measured by dripping it onto a digital refractometer.

#### Total acidity (g/L)

A sample of harvested grapes was crushed to extract the must. 10 mL of the filtered must was transferred to an Erlenmeyer flask, and approximately 20–30 mL of distilled water was added. Then, a few drops of phenolphthalein were added, and titration was performed with NaOH. The titration was stopped when the pH reached ≈ 8.1, and the total acidity was calculated. Titratable acidity (TA), expressed as g/L tartaric acid, was estimated using the Official OIV method [20].

#### Maturity index (%)

The maturity index was calculated by dividing the total acidity by 0.15 and then dividing by the soluble solids content (SSC).

#### Berry weight (mm)

This measurement was determined by weighing 10 randomly selected berries in grams using a Shimadzu precision balance at harvest time.

#### Berry length (mm)

The length of the grape berries was determined by measuring the length of 10 randomly selected berries using calipers with 0.1 mm accuracy.

#### Berry width (mm)

The berry width was determined by measuring the width of 10 randomly selected berries using calipers with 0.1 mm accuracy.

#### Peduncle detachment resistance (N)

This property was measured in grams using a dynamometer on 10 berries. Measurements were taken with a special device, which combines a dynamometer and a balance. It was measured by pulling the berry’s stem, which was hung by its stem, in the opposite direction to break it. Measured values are given in units of Newton (N).

#### Berry splitting resistance (N)

This property was calculated in grams using a dynamometer on 10 berries. Measurements were taken using a special device, a combination of a dynamometer and a balance. Force was applied to the grape until it split open, and the force required to split it was recorded. Measured values are given in units of Newton (N).

#### Specific taste

This measurement was performed according to the OIV 236 scale. Sensory tests were conducted on each genotype, and the specific taste characteristic was determined accordingly. The genotype was scored as follows: 1 if no specific aroma was present; 2 if muscat aroma was present; 3 if foxy aroma was present; 4 if herbaceous aroma was present; and 5 if any other aroma was present.

#### Berry skin colour

For colour measurement, thirty berries, three replicates of ten berries, were chosen randomly from different parts of the Cluster at the technological ripening period. Each berry is cleaned with a paper tissue. The colour values, L*, a*, b* were determined by a reflectance spectrophotometer Minolta CR-410 Chroma Meter (Minolta Corp., Osaka, Japan). Standard illuminant C was used as reference. Three measurements were made around the equatorial belt of each berry. The chroma value (C) was calculated as C =[(a*2 + b*2)]0.5 and hue (h) value as h = arctang b*/a*.

#### Uniformity of berry skin colour

Scoring was done by looking at the uniformity of the outer skin colours of the harvested grape berries. In this parameter, the OIV 226 scale was used to score from not uniform (1) and uniform (2).

#### Skin colour of berry

This measurement was made by evaluating the berries of ripening clusters using the OIV 225 scale (Table 4).

**Table 4:** Berry skin colour values according to OIV 225 scale values

Green yellow	1
Rose	2
Red	3
Grey	4
Dark Red Violet	5
Blue Black	6

#### Berry shape

This measurement was determined from berries randomly taken from the middle of the Cluster that maintained their structural integrity, considering the OIV 223 scale (Table 5).

**Table 5:**
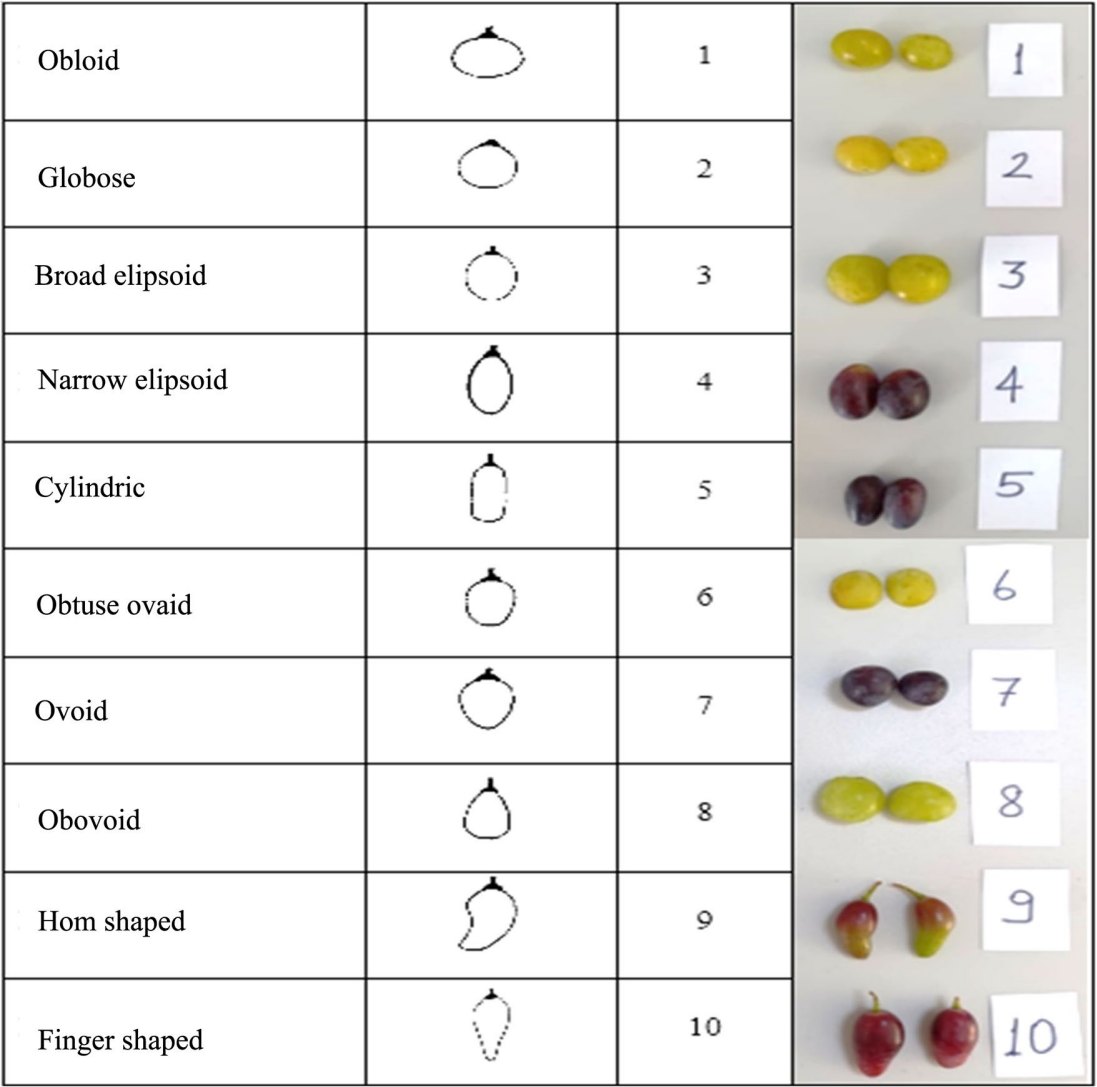
Berry shape scale values according to the OIV 223 scale

### Seed structure and seed status

#### Number of seeds

Seeds were extracted from 10 randomly selected clusters in three replicates and counted. The average of the resulting seed counts was then used to determine the seed count of the hybrids.

#### Single seed dry weight(mg)

Seeds extracted from 10 randomly selected clusters in three replicates from Clusters of each hybrid genotype were dried in an oven at 60 °C for 2 days. The dry weight of the seeds was then measured using a precision balance. The measured dry weight was divided by the number of seeds, and the dry seed weight was expressed in mg [18]. For a hybrid to be considered “seedless,” its dry seed weight must be less than 20 mg [21].

#### Veraison

The veraison parameter for hybrid genotypes was considered when softening and yellowing of the berries in the Clusters were observed for white varieties. For coloured varieties, data were collected based on the date when the berries in the Clusters began to colour.

#### Harvest date

For hybrid genotypes, harvest dates were determined based on maturity index values. The hybrid genotypes were harvested when their maturity index value reached approximately 20.

#### Sensory analyses

After harvest, sensory analysis of hybrids and varieties was performed by a panel of 6 members using the Atak and Kahraman [21] methods, with small modifications. The panelists who conducted the tasting tests comprised an expert team that had conducted breeding and tasting studies on hybrid grapes for many years. The list of sensory terms included descriptors of the general acceptance of the cluster (seeded/seedless, skin, flesh, homogeneity of colour), visual appearance (colour, berry shape/size, free disease/pest), odor (fruity/foxy, muscat, special), and taste (sweet, bitter, and sour/acidic). The scores were totaled to give a maximum score of 20 points, in terms of 4 criteria (Table 6).

**Table 6:** Criteria and scores used in sensory analysis (tasting) evaluation

General Appearance	Visual Appearance (Shape, Colour and Size Uniformity of Berries)	Odour	Taste	TOTAL
0–4	0–5	0–6	0–5	0–20

### Disease evaluations

#### Evaluation of *Botrytis cinereae*

This assessment was made according to the definition in criterion OIV-459 at harvest time. The assessment was made based on the visually observable Botrytis cinereae influence on the harvested Clusters. Scoring was based on 3 for low disease symptoms, 5 for moderate disease symptoms, and 7 for severe disease symptoms.

#### Evaluation of powdery mildew (*Uncinula necator*)

This assessment was made based on criterion OIV-456 at harvest time. The harvested grape clusters were evaluated for visible signs of powdery mildew. Scoring was based on 3 for low disease symptoms, 5 for moderate disease symptoms, and 7 for severe disease symptoms.

### Phenolic compound analyses

Analyses were performed using 13 selected hybrid genotypes with sufficient cluster counts and 4 reference varieties. In this part of the study, three clusters were selected from each of the 13 hybrid genotypes; after being washed and cleaned, the berries were separated from the cluster skeleton. To extract hybrid genotypes, hybrid grape berries were homogenized and crushed. This process was carried out so that all grape berries (skin, flesh, and seeds together) were thoroughly broken. After the peel, pulp, and seeds (seed traces) were homogenized to ensure thorough breakdown, they were divided into three 50 g portions, and analyses were carried out on the extracted samples in 3 replications. Methanol was used as the solvent for grape extraction.

#### Total phenolic content

In grape samples subjected to extraction procedures, the total phenolic content was determined in terms of gallic acid equivalent (mg/kg) using the Folin-Ciocalteu method reported by Waterhouse [22]. First, 40 µL of the extract was added to spectrophotometer cuvettes, followed by 3.16 mL of distilled water and 200 µL of Folin-Ciocalteu solution (Merck, Germany). After 2–3 min, 600 µL of sodium carbonate (Merck, Germany) solution (200 g/L) was added, and the mixture was left to stand at room temperature for 2 h. At the end of this period, absorbance values were read at 765 nm in a spectrophotometer (UV-Mini 1240, Shimadzu) against a control sample prepared with distilled water instead of the extract. The results are expressed as mg of gallic acid/mL (Table 11).

#### Total anthocyanin content

In colored grapes subjected to extraction processes, monomeric anthocyanins were determined using the pH-differential method and expressed as malvidin-3-glucoside (mg/kg) [23]. For the determination of total anthocyanins, extracts prepared with methanol were used in fresh grape samples, and extracts prepared with acetone were used in grape samples (Table 11). For this purpose, potassium chloride buffer (pH 1.0) and sodium acetate buffer (pH 4.5) solutions were prepared and mixed with the samples in the ratios determined in preliminary experiments, and allowed to equilibrate for a period of time (30 min). At the end of this period, the absorbances of both buffer solution samples were read at 520 nm and 700 nm wavelengths using a spectrophotometer. The following equation was used to determine the anthocyanin content of the samples.

$$\text A=(\text A520-\text A700)\mathrm{pH}1.0-(\text A520-\text A700)\mathrm{pH}4.5$$

$$\mathrm{Total}\;\mathrm{anthocyanin}\;\mathrm{content}\;(\mathrm{mg}/\text L)=((\text A)(\mathrm{MW})(\mathrm{Sf})(1000))/((\epsilon)\;\text l)$$

A: Absorbance difference, MW: Malvidin-3-glucoside molecular weight: 493.5, Sf: Dilution factor, ɛ: Molar absorption coefficient for Malvidin-3-glucoside: 28,000, l: Cuvette layer thickness: 1.

#### Antioxidant activity determination

Antioxidant activity in grapes subjected to extraction processes was measured using two methods.

#### (DPPH Free radical scavenging activity)

The total free radical scavenging capacity was determined using the DPPH method described by Brand-Williams et al. [24] and the results were given in mmol/L Trolox/kg. A decrease in the absorbance of the reaction mixture indicates a high free radical-scavenging activity. Different amounts of extracts (25, 50, 75 µL) were added to 1.95 mL of DPPH solution. After vortex mixing, it was left in the dark at room temperature for 30 min. Absorbances were read at 517 nm. % inhibition values were calculated according to the following equation.

$$\%\;\mathrm{Inhibition}\;=\;\lbrack(\mathrm{Acontrol}\;-\;\mathrm{Asample}/\;\mathrm{Acontrol})\rbrack\;\text x\;100$$

$$\mathrm{Acontrol}=\text{Absorbance of the control},$$

$$\mathrm{Asample}=\text{Absorbance of the sample}$$

Total antioxidant activity of pomace (on a dw basis) was also expressed in µMol/g of TEAC.

#### ABTS Radical scavenging activity

The ABTS method was performed as described by Re et al. [25]. The ABTS radical was formed by the reaction between a 7 mM ABTS solution and a 2.45 mM potassium persulfate solution. It was left in the dark at room temperature for 12 h. Before use, it was diluted with phosphate buffer (0.1 M, pH = 7.4) to achieve an absorbance of 0.7 ± 0.025 at 734 nm. 1 mL of ABTS solution was added to 3 mL of standard solutions (50–250 µg/mL, ethanol-methanol). After 30 min, the absorbance was read at 734 nm. The percentage inhibition values were calculated according to the following equation.

$$\%\;\mathrm{Inhibition}\;=\;\lbrack(\mathrm{Acontrol}\;-\;\mathrm{Asample}/\;\mathrm{Acontrol})\rbrack\;\text x\;100$$

$$\mathrm{Acontrol}=\text{Absorbance of the control},$$

$$\mathrm{Asample}=\text{Absorbance of the sample}$$

Total antioxidant activity of the pomace (on a dw basis) was expressed in µMol/g of TEAC (Trolox equivalent antioxidant capacity).

#### Statistical analysis

The SAS (JMP 18.1) software was used to statistically analyze the obtained data, and ANOVA (Analysis of Variance) was used to determine significant differences. The least significant difference (LSD) method was used to determine the significance levels for all accessions. Differences were considered significant at *p* < 0.05. Correlation coefficients (R) from a parametric Pearson’s test were calculated to evaluate covariance relationships among variables.

## Results

**Fig. 3:**
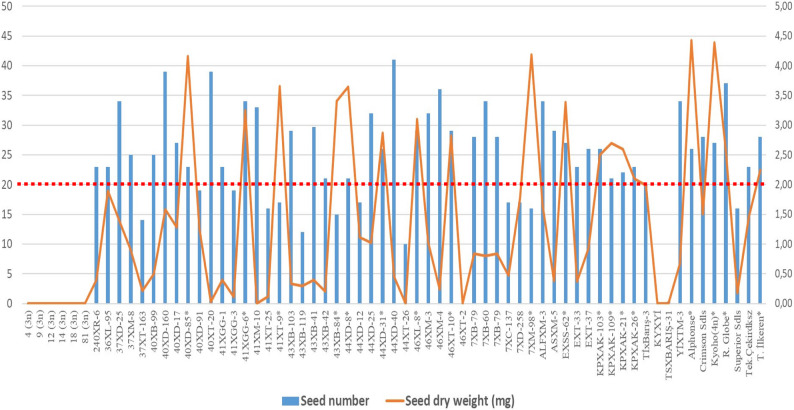
The representation of data on seed number and seed dry weight of hybrid genotypes and reference varieties in a clustered bar graph.( The red dashed line indicates hybrid genotypes with a dry seed weight below 20 mg, which are considered seedless)

**Fig. 4:**
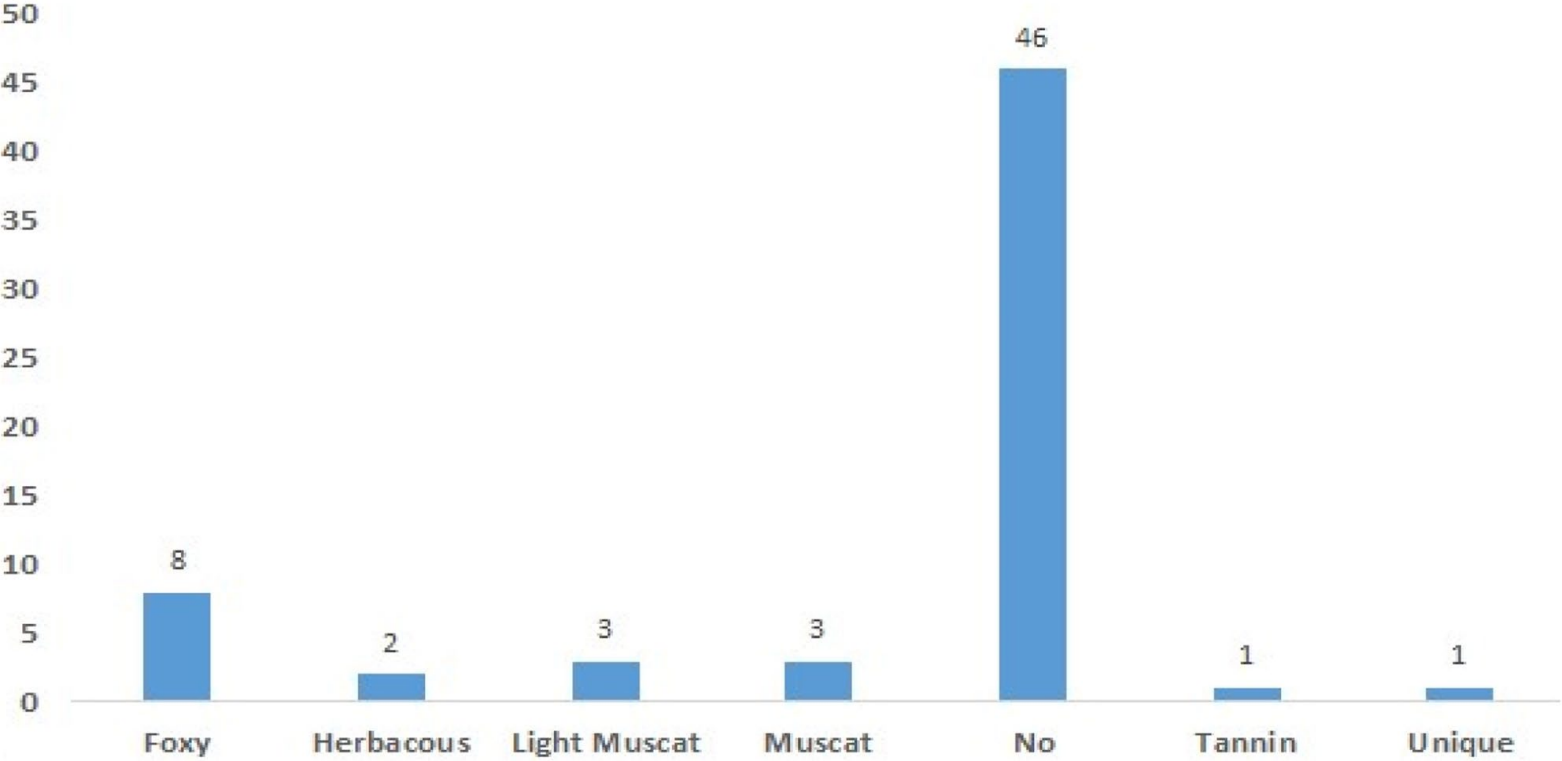
Graphical representation of the number of genotypes/varieties in terms of special taste

At the Tekirdağ Viticulture Research Institute, located in Tekirdağ province, the important quality characteristics and resistance to powdery mildew and downy mildew of the genotypes and reference varieties in the Phase 2 hybridization breeding plot were examined, and the results obtained are given in detail in Tables 7, 8, 9, 10 and 11, Figs. 3 and 4.

**Table 7:** Data on cluster characteristics of hybrid and reference varieties

Combination Code	Cluster weight (g)	Cluster density (1–7)	Number of berries per cluster	Berry Splitting Resistance (*N*)	Peduncle Detachment Resistance (*N*)
4	-	-	-	-	-
9	139.67^y^	3.00 ^f^	32.67 ^a−1^	-	-
12	-	3.67^ef^	-	-	-
14	-	-	-	-	-
18	-	-	-	-	-
81	-	-	-	-	-
240XR-6	439.00 ^n−r^	3.00 ^f^	117.67 ^n−v^	1250.4 ^l−n^	211.9 ^s−v^
36XL-95	707.33 ^e−f^	7.00 ^a^	169.00 ^f−j^	803.5 ^z−b1^	195.3 ^v−y^
37XD-25	457.33 ^l−p^	3.67^ef^	209.67 ^c−e^	1009.9 ^s−w^	208.6 ^s−v^
37XM-8	452.00 ^m−p^	3.67^ef^	108.00 ^q−y^	1110.3 ^o−s^	208.8 ^s−v^
37XT-163	822.00 ^bc^	5.00 ^cd^	245.00 ^b−c^	654.0 ^c1^	104.0 ^b1^
40XB-99	450.00 ^m−p^	7.00 ^a^	197.00 ^d−f^	868.6 ^x−a1^	229.1 ^o−p^
40XD-160	673.67 ^e−f^	5.00 ^cd^	156.33 ^h−m^	869.0 ^x−a1^	318.6 ^g−ı^
40XD-17	858.33 ^b^	5.00 ^cd^	174.00 ^e−i^	1121.4 ^o−s^	222.6 ^o−t^
40XD-85*	504.67 ^ı−m^	3.00 ^f^	92.00 ^t−z^	1339.6 ^l−k^	329.6 ^g^
40XD-91	266.67 ^w−x^	3.00 ^f^	102.67 ^r−y^	518.5 ^e1^	276.4 ^s−v^
40XT-20	450.33 ^m−p^	3.00 ^f^	152.00 ^h−n^	970.1 ^u−x^	162.0 ^z−a1^
41XGG-1	562.67 ^h−i^	5.00 ^cd^	171.33 ^f−j^	1049.5 ^r−v^	394.0 ^e^
41XGG-3	653.00 ^f−g^	3.00 ^f^	168.00 ^f−j^	668.9 ^c1^	38.2 ^c1^
41XGG-6*	552.00 ^h−i^	3.67^ef^	80.00 ^w−z^	1855.6 ^e^	283.3 ^k−m^
41XM-10	448.33 ^m−p^	5.00 ^cd^	110.33 ^p−y^	1141.4 ^n−p^	234.5 ^o−p^
41XT-25	383.33 ^q−u^	3.67^ef^	83.00 ^v−z^	1689.6 ^h−ı^	461.0 ^c^
41XT-9*	473.33 ^k−n^	3.67^ef^	136.67 ^j−s^	1687.1 ^ı^	448.8 ^c−d^
43XB-103	559.33 ^h−i^	5.67^bc^	410.33 ^a^	722.9 ^b1−c1^	189.5 ^x−y^
43XB-119	280.67 ^w−x^	3.00 ^f^	103.33 ^r−y^	1021.7 ^r−v^	264.9 ^m−n^
43XB-41	260.00 ^w−x^	3.00 ^f^	183.67 ^e−h^	955.0 ^v−x^	307.2 ^h−j^
43XB-42	443.33 ^m−q^	5.00 ^cd^	166.67 ^f−k^	795.0 ^z−b1^	110.7 ^b1^
43XB-84*	781.67 ^cd^	5.67^bc^	160.00 ^g−l^	2064.0 ^e^	383.1 ^e^
44XD-8*	666.67 ^e−g^	5.00 ^cd^	100.33 ^s−y^	1639.5 ^ı−j^	600.6 ^b^
44XD-12	379.33 ^r−v^	3.00 ^f^	258.67 ^b^	661.9 ^c1^	6.4 ^d1^
44XD-25	543.33 ^i−j^	7.00 ^a^	101.67 ^r−y^	1730.6 ^f−h^	445.1 ^c−d^
44XD-31*	396.67 ^p−t^	3.00 ^f^	77.33 ^x−z^	2268.5 ^d^	379.0 ^e^
44XD-40	438.67 ^n−r^	3.00 ^f^	146.67 ^i−p^	830.5 ^z−b1^	243.5 ^n−p^
44XT-26	550.00 ^h−i^	5.00 ^cd^	152.67 ^h−n^	999.7 ^u−w^	223.3 ^o−s^
46XL-8*	550.67 ^h−i^	5.00 ^cd^	101.67 ^r−y^	2781.4 ^c^	299.1 ^ı−k^
46XM-3	466.67 ^k−o^	5.67^bc^	163.33 ^f−k^	1409.5 ^k^	222.0 ^p−t^
46XM-4	409.33 ^o−t^	3.00 ^f^	166.67 ^f−k^	1133.3 ^o−r^	188.8 ^x−y^
46XT-10*	550.67 ^h−i^	7.00 ^a^	113.67 ^o−x^	836.4 ^y−b1^	202.9 ^s−x^
46XT-2	382.67 ^q−u^	1.67^g^	87.67 ^u−z^	972.9 ^u−x^	296.1 ^j−l^
7 X B-79	253.33 ^x^	5.00 ^cd^	114.33 ^o−w^	648.9 ^c1^	200.5 ^t−x^
7XB-60	690.67 ^e−f^	5.00 ^cd^	137.33 ^j−r^	1348.9 ^k−l^	116.2 ^b1^
7XB-79	285.00 ^w−x^	5.00 ^cd^	114.33 ^o−w^	648.9 ^c1^	200.5 ^t−x^
7XC-137	521.67 ^i−k^	4.3 ^de^	232.67 ^b−d^	345.1 ^f−1^	113.4 ^b1^
7XD-258	543.33 ^i−j^	5.00 ^cd^	238.67 ^b−c^	557.6 ^e1^	225.8 ^o−p^
7XM-98*	723.33 ^de^	5.67 ^bc^	171.33 ^f−j^	2245.7 ^d^	393.2 ^e^
ALFXM-3	322.00 ^u−w^	3.00 ^f^	87.33 ^u−z^	1181.0 ^n−p^	437.1 ^d^
ASXM-5	468.33 ^k−o^	5.00 ^cd^	157.67 ^h−l^	1418.1 ^k^	149.0 ^a1^
EXSS-62*	395.00 ^p−t^	3.00 ^f^	75.67 ^y−z^	1815.5 ^f^	381.7 ^e^
EXT-33	516.00 ^i−l^	5.00 ^cd^	120.67 ^m−u^	1003.5 ^u−w^	198.7 ^v−y^
EXT-37	566.67 ^h−ı^	5.00 ^cd^	137.33 ^j−r^	1195.9 ^n−p^	355.0 ^f^
KPXAK-103*	369.33 ^s−v^	3.67^ef^	119.33 ^n−v^	1109.1 ^o−s^	222.8 ^o−t^
KPXAK-109*	560.67 ^h−i^	4.33^de^	126.33 ^l−t^	1083.1 ^p−u^	177.0 ^y−z^
KPXAK-21*	319.00 ^v−w^	4.33^de^	194.33 ^e−g^	1203.1 ^m−o^	244.9 ^n−o^
KPXAK-26*	483.33 ^j−n^	4.33^de^	147 ^i−o^	965.5 ^v−x^	241.3 ^o−p^
KYXYİ	-	-	-	902.6 ^w−z^	226.7 ^o−p^
TİXBARIŞ-3	426.33^n−s^	3.00 ^f^	61.00 ^z−a1^	1801.5 ^f−g^	183.4 ^x−z^
TSXBARIŞ-31	400.00 ^p−t^	5.00 ^cd^	146.33 ^i−p^	947.5 ^v−y^	235.6 ^o−p^
Yİ X TM-3	355.00 ^t−v^	4.33^de^	130.67 ^k−s^	783.9 ^a1−b1^	355.6 ^f^
A. Lavallée*	549.67 ^h−i^	6.33 ^ab^	75.33 ^y−z^	1316.5 ^k−m^	224.8 ^o−s^
Crimson Seedlss	550.00 ^h−i^	5.00 ^cd^	135.00 ^j−s^	3406.7 ^a^	816.4 ^a^
Kyoho*	152.33^y^	1.00 ^g^	35.00 ^a−l^	1563.9 ^j^	220.7 ^s−t^
Red Globe	968.33 ^a^	5.00 ^cd^	145.67 ^i−p^	2193.1 ^d^	595.2 ^b^
Superior Seedlss	438.67 ^n−r^	5.00 ^cd^	126.67 ^l−t^	3022.4 ^b^	272.5 ^m^
Tek. Çekirdsz	289.67 ^w−x^	3.67^ef^	79.33 ^w−z^	835.9 ^y−b1^	326.5 ^g−h^
T.İlkeren*	608.33^gh^	6.33^ab^	142 ^i−q^	1708.5 ^g−ı^	229.4 ^o−p^
*LSD(0.01)*	*63.16*	*1.13*	*36.63*	*113.15*	*22.31*

* Seeded genotypes/reference varieties

Superscript letters indicate that there are significant differences at least at the *p* < 0.05 level in the relevant quality characteristics among hybrid genotypes/reference varieties

**Table 8:** Data on berry and quality characteristics of hybrid and reference varieties

Combination code	Berry weight (g)	Berry width(mm)	Berry lenght(mm)	Uniformity of berry colour	TSS (%)	Total acidity (g/L)	Maturity Index
4	3.74 ^v^	17.30 ^p−s^	21.3 ^h−r^	1	24.8 ^a−b^	6.0 ^e−f^	27.78 ^j−r^
9	3.33 ^y−z^	17.20 ^q−t^	18.9 ^m−x^	2	15.0 ^w−c1^	5.6 ^f−g^	18.09 ^x−b1^
12	4.33 ^r−s^	18.80 ^ı−m^	22.2 ^h−n^	1	19.0 ^f−j^	3.9 ^o−r^	33.31 ^f−ı^
14	4.16 ^s−t^	18.60 ^j−n^	20.2 ^ı−u^	1	19.2 ^f−j^	-	-
18	3.44 ^x−y^	17.13 ^q−t^	19.8 ^ı−u^	1	21.0 ^d^	-	-
81	2.09 ^ı1^	15.00 ^y−a1^	15.4 ^w−z^	2	15.6 ^s−b1^	-	-
240XR-6	4.83 ^n−p^	15.40 ^x−z^	28.2 ^a−c^	1	16.5 ^o−x^	3.9 ^o−r^	28.34 ^ı−q^
36XL-95	4.65 ^p−q^	19.26 ^g−k^	22.1 ^h−n^	2	18.9 ^f−j^	4.6 ^h−n^	27.37 ^k−t^
37XD-25	2.67 ^e1−f1^	16.20 ^t−x^	17.1 ^s−z^	1	16.4 ^o−x^	3.6 ^q−t^	30.4 ^ı−n^
37XM-8	4.46 ^q−r^	18.50 ^j−n^	22.0 ^h−p^	1	17.3 ^ı−u^	4.3 ^j−p^	27.31 ^k−t^
37XT-163	4.40 ^r^	18.50 ^j−n^	22.5 ^e−m^	2	14.3 ^a1−c1^	3.0 ^t−v^	31.75 ^g−l^
40XB-99	2.77 ^d1−e1^	16.80 ^r−u^	15.6 ^w−z^	1	17.7 ^g−s^	6.0 ^e−f^	19.8 ^v−a1^
40XD-160	4.63 ^p−q^	20.26 ^d−g^	20.9 ^h−s^	1	15.4 ^t−c1^	4.0 ^m−r^	25.68 ^n−u^
40XD-17	5.78 ^g^	20.20 ^e−g^	24.2 ^d−h^	1	16.1 ^q−a1^	5.0 ^g−ı^	22.19 ^s−y^
40XD-85*	6.79 ^d^	22.03 ^b^	23.5 ^d−ı^	1	16.3 ^p−x^	2.9 ^u−v^	37.95 ^d−f^
40XD-91	5.20 ^j−l^	20.00 ^f−h^	22.2 ^h−n^	1	18.3 ^g−q^	3.5 ^r−u^	35.83 ^e−h^
40XT-20	2.37 ^g1^	15.50 ^x−z^	17.1 ^s−z^	1	16.6 ^o−x^	4.7 ^h−l^	23.54 ^q−w^
41XGG-1	4.18 ^s−t^	19.30 ^g−ı^	19.2 ^l−w^	1	16.9 ^o−x^	5.03 ^g−ı^	22.58 ^r−x^
41XGG-3	3.76 ^u−v^	18.00 ^m−q^	19.2 ^l−w^	1	16.5 ^o−x^	3.6 ^q−t^	31.22 ^g−m^
41XGG-6*	8.66 ^a^	25.10 ^a^	24.9 ^c−f^	1	15.1 ^u−c1^	2.8 ^v^	36.54 ^d−g^
41XM-10	5.57 ^g−h^	21.10 ^b−e^	22.0 ^h−p^	1	15.9 ^r−b1^	4.4 ^ı−o^	24.17 ^o−v^
41XT-25	4.87 ^n−o^	19.10 ^h−l^	22.8 ^e−m^	1	15.4 ^t−c1^	4.5 ^ı−o^	23.32 ^q−x^
41XT-9*	6.57 ^e^	22.00 ^b^	23.2 ^d−ı^	2	15.9 ^r−b1^	4.8 ^h−k^	22.^21 s−y^
43XB-103	1.42 ^k1^	13.06 ^c1−d1^	14.9 ^y−z^	2	20.8 ^d−f^	4.2 ^k−q^	33.14 ^f−j^
43XB-119	3.12 z-^b1^	15.50 ^x−z^	14.2 ^z^	2	18.4 ^g−p^	7.5 ^c^	16.4 ^a1−b1^
43XB-41	1.67 ^j1^	13.80 ^b1−c1^	15.4 ^w−z^	1	21.0 ^d^	4.6 ^h−n^	30.43 ^h−n^
43XB-42	3.14 ^z−b1^	17.40 ^o−s^	18.1 ^p−z^	1	19.8 ^f−g^	4.67 ^h−m^	28.36 ^ı−q^
43XB-84*	5.09 ^k−m^	19.40 ^g−ı^	22.9 ^e−l^	1	19.7 ^f−g^	4.0 ^m−r^	32.82 ^f−j^
44XD-8*	7.19 ^c^	20.00 ^f−h^	29.6 ^a^	1	13.3 ^c1^	3.7 ^p−s^	23.94 ^p−w^
44XD-12	2.11 ^h1−ı1^	14.80 ^z−a1^	16.8 ^t−z^	1	17.0 ^j−w^	8.07 ^b−c^	14.03 ^b1−c1^
44XD-25	4.78 ^o−p^	19.40 ^g−ı^	22.33 ^f−n^	1	16.8 ^o−x^	8.5 ^b^	13.3 ^b1−c1^
44XD-31*	6.72 ^d−e^	18.30 ^k−p^	20.5 ^h−t^	1	14.7 ^x−c1^	3.0 ^t−v^	32.91 ^f−j^
44XD-40	3.74 ^v^	19.40 ^g−ı^	19.5 ^l−u^	1	8.9 ^d1^	4.5 ^ı−o^	13.26 ^b1−c1^
44XT-26	4.18 ^s−t^	19.20 ^g−k^	21.5 ^h−p^	1	13.9 ^a1−c1^	5.0 ^g−ı^	18.57 ^w−b1^
46XL-8*	5.56 ^h^	21.30 ^b−d^	23.0 ^e−ı^	1	19.1 ^f−j^	3.5 ^r−u^	36.32 ^e−g^
46XM-3	2.95 ^b1−d1^	16.60 ^s−w^	16.8 ^t−z^	1	16.7 ^o−x^	4.6 ^h−n^	24.24 ^o−v^
46XM-4	2.52 ^f1−g1^	16.00 ^u−y^	17.8 ^r−z^	1	18.6 ^f−o^	5.0 g-ı	25.15 ^n−v^
46XT-10*	5.32 ^ı−j^	17.76 ^m−r^	21.5 ^h−p^	1	17.2 ^ı−u^	4.4 ^ı−o^	26.04 ^m−u^
46XT-2	4.97 ^m−o^	20.53 ^c−f^	20.3 ^h−u^	1	18.9 ^f−j^	3.93 ^n−r^	32.18 ^g−k^
7XB-79	3.17 ^z−a1^	16.60 ^s−w^	19.0 ^l−x^	1	17.1 ^j−u^	3.9 ^o−r^	29.44 ^ı−o^
7XB-60	5.04 ^l−n^	19.40 ^g−ı^	20.2 ^ı−u^	1	20.7 ^d−f^	3.47 ^r−v^	39.79 ^d−e^
7XB-79	3.17 ^z−a1^	16.60 ^s−w^	19.0 ^l−x^	1	17.1 ^j−u^	3.9 ^o−r^	29.24 ^ı−p^
7XC-137	2.31 ^g1−h1^	15.66 ^v−z^	17.4 ^r−z^	1	16.3 ^p−x^	6.5 ^d−e^	16.71 ^z−b1^
7XD-258	3.57 ^v−x^	18.10 ^l−q^	18.4 ^n−y^	1	16.8 ^o−x^	5.0 ^g−ı^	22.6 ^r−x^
7XM-98*	6.23 ^f^	19.80 ^f−ı^	28.5 ^a−c^	1	22.4 ^c−d^	4.5 ^ı−o^	33.42 ^f−ı^
ALFXM-3	3.68 ^v−w^	18.40 ^j−o^	18.9 ^m−x^	1	16.5 ^o−x^	5.0 ^g−ı^	21.98 ^t−z^
ASXM-5	3.04 ^a1−c1^	15.60 ^w−z^	23.0 ^e−ı^	1	17.2 ^ı−u^	3.9 ^o−r^	29.91 ^ı−n^
EXSS-62*	7.50 ^b^	21.90 ^b^	22.1 ^h−n^	1	13.7 ^b1−c1^	4.6 ^h−n^	19.99 ^v−a1^
EXT-33	6.63 ^d−e^	20.60 ^c−f^	26.3 ^a−f^	2	16.6 ^o−x^	4.03 ^l−r^	27.41 ^k−s^
EXT-37	6.15 ^f^	22.00 ^b^	21.4 ^h−p^	2	19.0 ^f−j^	4.3 ^j−p^	30.2 ^ı−n^
KPXAK-103*	3.5 ^w−y^	14.30 ^a1−b1^	28.9 ^a−b^	1	22.6 ^b−d^	3.1 ^s−v^	48.58 ^a−b^
KPXAK-109*	4.45 ^q−r^	17.53 ^n−s^	24.2 ^d−h^	1	18.3 ^g−q^	2.97 ^t−v^	41.77 ^c−d^
KPXAK-21*	2.45 ^g1^	12.43 ^d1^	27.1 ^a−d^	1	25.5 ^a^	3.2 ^s−v^	53.13 ^a^
KPXAK-26*	3.97 ^t−u^	15.13 ^x−a1^	29.2 ^a^	1	23.9 ^a−c^	3.5 ^r−u^	45.54 ^b−c^
TİXBARIŞ-3	4.94 ^m−o^	21.40 ^b−c^	20.5 ^h−t^	1	17.2 ^ı−u^	6.7 ^d^	17.11 ^y−b1^
KYXYİ	6.13 ^f^	19.20 ^g−k^	26.3 ^a−f^	2	22.8 ^b−d^	6.0 ^e−f^	25.4 ^n−u^
TSXBARIŞ-31	3.70 ^v−w^	17.20 ^q−t^	16.5 ^u−z^	1	17.5 ^ı−t^	12 ^a^	9.72 ^c1^
YİXTM-3	2.85 ^c1−e1^	15.80 ^u−z^	18.4 ^n−y^	1	16.5 ^o−x^	3.7 ^p−s^	29.72 ^ı−n^
A. Lavallée*	8.80 ^a^	24.66 ^a^	25.1 ^b−f^	1	16.8 ^o−x^	3.07 ^s−v^	36.49 ^d−g^
Crimson Seedlss	4.01 ^t^	16.70 ^r−v^	23.4 ^d−ı^	1	19.4 ^f−ı^	4.9 ^g−j^	26.42 ^l−u^
Kyoho(4n)*	5.44 ^h−ı^	20.13 ^e−h^	22.6 ^e−m^	1	18.8 ^f−j^	4.2 ^k−q^	29.81 ^ı−n^
Red Globe*	8.87 ^a^	24.10 ^a^	26.4 ^a−e^	1	14.2 ^a1−c1^	3.7 ^p−s^	25.57 ^n−u^
Sup. Seedless	4.52 ^q−r^	18.73 ^ı−m^	20.0 ^ı−u^	1	18.0 ^g−r^	5.23 ^g−h^	22.91 ^r−x^
Tek.Çekirdeksizi	4.00 ^t^	18.73 ^ı−m^	18.3 ^p−y^	1	19.2 ^f−j^	3.4 ^r−v^	37.62 ^d−f^
Trakya İlkeren*	5.29 ^ı−k^	16.70 ^r−v^	15.1 ^x−z^	1	14.8 ^w−c1^	4.63 ^h−m^	21.66 ^u−a1^
*LSD(0.01)*	*0.22*	*1.09*	*3.97*	*-*	*2.24*	*0.69*	*5.41*

* Seeded genotypes/reference varieties

Superscript letters indicate that there are significant differences at least at the *p* < 0.05 level in the relevant quality characteristics among hybrid genotypes/reference varieties

**Table 9:** Data on seed and berry colour hybrid and reference varieties

Combination Code	Seed number	Seed dry weight (mg)	L*	a*	b*	Hue	Chroma
4	0 ^s^	0 ^z^	31.12 ^s−w^	-0.08^t−y^	− 2.86 ^o−s^	2.86 ^r−a1^	268.69 ^a−c^
9	0 ^s^	0 ^z^	30.96 ^s−w^	1.02 ^h−o^	-2.33 ^o−r^	2.60 ^v−c1^	293.14 ^a−b^
12	0 ^s^	0 ^z^	30.85 ^s−x^	-0.2 ^t−y^	-2.26 ^o−r^	2.27 ^x−c1^	265.07 ^b−c^
14	0 ^s^	0 ^z^	29.84 ^u−x^	0.45 ^l−u^	-1.54 ^m−o^	1.62 ^c1^	286.69 ^a−c^
18	0 ^s^	0 ^z^	39.95 ^g−j^	-1.17 ^b1−e1^	7.37 ^c−d^	7.46 ^de^	99.09 ^d−f^
81	0 ^s^	0 ^z^	37.26 ^k−n^	0.57 ^k−t^	-2.42 ^o−r^	2.49 ^v−c1^	283.23 ^a−c^
240XR-6	2.3 ^ı−l^	3.95 ^y−z^	28.53 ^w−x^	3.69 ^b^	-0.51 ^m^	3.75 ^k−s^	231.78 ^c^
36XL-95	2.3 ^ı−l^	19 ^l−o^	32.12 ^q−u^	0.82 ^ı−r^	-2.02 ^n−q^	2.21 ^z−c1^	293.82 ^a−b^
37XD-25	3.4 ^b−d^	13.91 ^o−s^	31.95 ^q−u^	0.73 ^j−s^	-2.42 ^o−r^	2.54 ^v−c1^	287.18 ^a−c^
37XM-8	2.5 ^g−k^	8.96 ^s−v^	32.93 ^p−t^	0.23 ^o−x^	-2.98 ^p−s^	2.99 ^q−a1^	274.5 ^a−c^
37XT-163	1.4 ^p−r^	2.14 ^z^	33.82 ^o−r^	1.35 ^f−k^	-2.48 ^o−r^	2.93 ^r−a1^	300.26 ^a−b^
40XB-99	2.5 ^g−k^	4.96 ^v−z^	32.65 ^q−t^	1.6 ^f−ı^	-1.95 ^n−q^	2.59 ^v−c1^	309.98 ^a−b^
40XD-160	3.9 ^ab^	15.82 ^m−q^	39.78 ^g−k^	-0.05 ^s−y^	4.97 ^f−g^	4.99 ^g−j^	92.15 ^d−f^
40XD-17	2.7 ^f−ı^	12.81 ^p−t^	43.88 ^b−e^	-1.92 ^e1−g1^	6.49 ^c−d^	6.77 ^e−f^	106.47 ^d−f^
40XD-85*	2.3 ^ı−l^	41.56 ^a−b^	47.48 ^a^	1.42 ^f−j^	2.51 ^j−l^	3.00 ^q−a1^	55.51 ^e−f^
40XD-91	1.9 ^l−p^	12.67 ^p−t^	41.94 ^e−g^	-0.41 ^w−b1^	2.31 ^k−l^	2.49 ^v−c1^	109.91 ^d−e^
40XT-20	3.9 ^ab^	0.3 ^z^	36.32 ^m−o^	1.6 ^f−ı^	-4.01 ^s−t^	4.32 ^h−p^	291.62 ^a−b^
41XGG-1	2.3 ^ı−l^	3.95 ^y−z^	40.71 ^f−h^	3.17 ^b−c^	4.03 ^f−ı^	5.63 ^f−g^	50.05 ^f^
41XGG-3	1.9 ^l−p^	1.15 ^z^	32.10 ^q−u^	2.95 ^b−c^	-1.97 ^n−q^	3.55 ^m−v^	326.5 ^a^
41XGG-6*	3.4 ^b−d^	32.52 ^c−f^	33.39 ^p−s^	1.59 ^f−ı^	-2.90 ^o−s^	3.34 ^n−z^	300.04 ^a−b^
41XM-10	3.3 ^c−e^	0.03 ^z^	34.02 ^o−q^	2.13 ^d−f^	-2.41 ^o−r^	3.30 ^o−z^	312.96 ^a−b^
41XT-25	1.6 ^o−q^	1.19 ^z^	33.35 ^p−s^	2.9 ^b−d^	-2.70 ^o−s^	4.13 ^ı−q^	316.18 ^a−b^
41XT-9*	1.7 ^m−q^	36.52 ^b−c^	44.60 ^b−d^	-0.46 ^x−b1^	4.29 ^f−ı^	4.32 ^h−p^	96.39 ^d−f^
43XB-103	2.9 ^d−g^	3.34 ^y−z^	34.16 ^o−q^	0.34 ^m−w^	-3.28 ^q−s^	3.33 ^n−z^	276.91 ^a−c^
43XB-119	1.2 ^qr^	3.0 ^y−z^	38.87 ^h−m^	-1.03 ^z−d1^	4.66 ^f−h^	4.77 ^g−l^	102.58 ^d−f^
43XB-41	2.97 ^d−g^	4.0 ^y−z^	38.41 ^h−m^	0.59 ^k−t^	-4.83 ^t^	4.90 ^g−k^	277.68 ^a−c^
43XB-42	2.1 ^k−m^	2.0 ^z^	42.19 ^d−g^	-1.89 ^e1−g1^	4.02 ^f−ı^	4.47 ^g−n^	116.98 ^d^
43XB-84*	1.5 ^o−r^	34.06 ^c−d^	28.94 ^v−x^	0.87 ^ı−q^	-1.57 ^m−o^	1.80 ^c1^	299.96 ^a−b^
44XD-8*	2.1 ^k−m^	36.42 ^b−c^	45.83 ^a−b^	-2.35 ^f1−h1^	6.37 ^d−e^	6.79 ^e−f^	110.27 ^d−e^
44XD-12	1.7 ^m−q^	11.17 ^q−u^	45.19 ^a−c^	-2.87 ^h1^	11.50 ^b^	11.85 ^b^	103.96 ^d−f^
44XD-25	3.2 ^c−f^	10.25 ^r−u^	40.73 ^f−h^	-1.87 ^e1−g1^	5.11 ^e−f^	5.45 ^g−h^	110.11 ^d−e^
44XD-31*	2.6 ^gı^	28.69 ^e−h^	40.03 ^g−j^	-1.86 ^e1−g1^	7.74 ^c^	7.97 ^d^	103.65 ^d−f^
44XD-40	4.1 ^a^	4.5 ^v−z^	39.11 ^h−l^	-1.01 ^z−d1^	4.98 ^f^	5.10 ^g−ı^	103.65 ^d−f^
44XT-26	1.0 ^r^	0 ^z^	38.50 ^h−m^	-1.64 ^d1−f1^	1.75 ^l^	2.42 ^v−c1^	133.81 ^d^
46XL-8*	2.9 ^d−g^	31 ^d−g^	37.67 ^j−n^	0.2 ^p−x^	3.43 ^h−k^	3.44 ^m−w^	86.88 ^d−f^
46XM-3	3.2 ^c−f^	10.28 ^r−u^	35.53 ^n−p^	1.03 ^g−n^	-3.45 ^r−s^	3.65 ^l−s^	288.01 ^a−c^
46XM-4	3.6 ^a−c^	2.44 ^z^	36.85 ^l−n^	-0.83 ^y−c1^	3.29 ^ı−k^	3.39 ^n−y^	104.17 ^d−f^
46XT-10*	2.9 ^d−g^	28.27 ^f−h^	37.69 ^ı−n^	-0.29 ^u−z^	3.89 ^f−ı^	3.91 ^j−r^	94.05 ^d−f^
46XT-2	0 ^s^	0 ^z^	34.18 ^o−q^	0.92 ^ı−p^	-3.29 ^q−s^	3.42 ^n−x^	285.34 ^a−c^
7XB-79	2.8 ^e−g^	8.42 ^t−v^	43.766 ^b−e^	-1.9 ^e1−g1^	6.51^c−d^	6.78 ^e−f^	106.27 ^d−f^
7XB-60	3.4 ^b−d^	8.05 ^t−y^	38.674 ^h−m^	-1.14 ^a1−e1^	5.12^e−f^	5.25 ^g−ı^	103.04 ^d−f^
7XB-79	2.8 ^e−g^	8.42 ^t−v^	43.766 ^b−e^	-1.9 ^e1−g1^	6.51^c−d^	6.78 ^e−f^	106.27 ^d−f^
7XC-137	1.7 ^m−q^	4.7 ^v−z^	30.517 ^t−x^	0.41 ^l−v^	-2.21^o−r^	2.25 ^y−c1^	280.39 ^a−c^
7XD-258	1.7 ^m−q^	17.7 ^l−p^	40.30 ^g−ı^	-1.03 ^z−d1^	3.61 ^g−k^	3.77 ^k−s^	107.14 ^d−f^
7XM-98*	1.6 ^o−q^	41.87 ^a^	32.25 ^q−u^	1.77 ^e−h^	-1.94 ^n−q^	2.67 ^s−c1^	312.95 ^a−b^
ALFXM-3	3.4 ^b−d^	16.61 ^m−p^	33.18 ^p−s^	0.27 ^n−x^	-3 ^p−s^	3.01 ^q−a1^	275.24 ^a−c^
ASXM-5	2.9 ^d−g^	3.82 ^y−z^	37.58 ^j−n^	-0.36 ^v−a1^	4.32 ^f−ı^	4.36 ^h−o^	96.32 ^d−f^
EXSS-62*	2.7 ^f−ı^	33.85 ^c−e^	33.31 ^p−s^	1.1 ^g−m^	-2.81 ^o−s^	3.18 ^p−a1^	293.31 ^a−b^
EXT-33	2.3 ^ı−l^	3.65 ^y−z^	32.88 ^q−t^	2.11 ^d−f^	-1.95 ^n−q^	3 ^q−a1^	320.41 ^a−b^
EXT-37	2.6 ^g−ı^	9.26 ^s−v^	38.16 ^h−m^	2.53 ^c−e^	-2.96 ^p−s^	3.91 ^j−r^	310.65 ^a−b^
KPXAK-103*	2.6 ^g−ı^	25.07 ^h−k^	31.00 ^s−w^	0.1 ^q−x^	-2.37 ^o−r^	2.38 ^w−c1^	272.94 ^a−c^
KPXAK-109*	2.1 ^k−m^	26.95 ^g−ı^	31.62 ^q−u^	0.06 ^r−x^	-2.51 ^o−r^	2.51 ^v−c1^	271.68 ^a−c^
KPXAK-21*	2.2 ^ı−m^	25.95 ^g−j^	32.00 ^q−u^	1.14 ^g−l^	-2.4 ^o−r^	2.68s ^v−c1^	295.37 ^a−b^
KPXAK-26*	2.3 ^ı−l^	21 ^j−m^	30.78 ^s−x^	1.2 ^g−l^	-2.09 ^n−r^	2.43 ^v−c1^	300.45 ^a−b^
TİxBarış-3	2.0 ^k−o^	19.9 ^k−n^	43.04 ^c−f^	-2.5 ^g1−h1^	10.35 ^b^	10.68 ^c^	104.13 ^d−f^
KYXYİ	0 ^s^	0 ^z^	32.63 ^q−t^	1.09 ^g−m^	-1.65^m−p^	2.07 ^a1−c1^	306.6 ^a−b^
TSXBARIŞ-31	0.03 ^s^	0 ^z^	38.45 ^h−m^	-1.43 ^c1−e1^	3.85 ^f−j^	4.11 ^ı−q^	110.45 ^d−e^
YİXTM-3	3.4 ^b−d^	6.7 ^u−y^	43.20 ^c−f^	-1.8 ^d1−g1^	6.57 ^c−d^	6.82 ^d−e^	105.39 ^d−f^
Alphonse*	2.6 ^g−ı^	44.26 ^a^	32.11 ^q−u^	-0.09 ^t−y^	-3.16 ^q−s^	3.16 ^p−a1^	268.82 ^a−c^
Crimson Sdls	2.8 ^e−g^	15 ^n−r^	31.35 ^r−v^	4.94 ^a^	-0.73^m−n^	5.11 ^g−ı^	231.08 ^c^
Kyoho(4n)*	2.7 ^f−ı^	43.92 ^a^	31.81 ^q−u^	1.02 ^h−o^	-2.43 ^o−r^	2.69 ^s−c1^	293.93 ^a−b^
R. Globe*	3.7 ^a−c^	25.72 ^h−j^	39.04 ^h−l^	3.34 ^b^	-3.11 ^q−s^	4.6 ^g−m^	316.31 ^a−b^
Superior Sdls	1.6 ^o−q^	1.75 ^z^	47.77 ^a^	-4.32 ^ı−1^	14.02 ^a^	14.68 ^a^	107.16 ^d−f^
Tek.Çekirdkszzi	2.3 ^ı−l^	14.56 ^o−r^	34.02 ^o−q^	1.49 ^f−j^	-3.17 ^q−s^	3.51 ^m−w^	295.19 ^a−b^
T. İlkeren*	2.8 ^e−g^	22.42 ^ı−l^	28.29 ^x^	1.82 ^e−g^	-2.28 ^o−r^	2.94 ^r−a1^	308.85 ^a−b^
*LSD(0.01)*	*0.53*	*5.2*	*2.62*	*0.8*	*1.36*	*1.16*	*58.61*

* Seeded genotypes/reference varieties

Superscript letters indicate that there are significant differences at least at the *p* < 0.05 level in the relevant quality characteristics among hybrid genotypes/reference varieties

**Table 10:** Some important traits and disease evaluation of hybrids and reference varieties

Combination Code	Special taste	Skin colour	Veeraison date	Harvest date	Degustation score	B.cinerea score (3/5/7)	Powdery mildew score (3/5/7)
4	Foxy	6	15 Jul.	11 Agu.	-	3^c^	3
9	Foxy	4	10 Jul.	15 Sep.	-	3^c^	3
12	Foxy	6	15 Jul.	19 Agu.	-	3^c^	3
14	Foxy	6	22 Jul.	25 Jul.	-	3^c^	7
18	Foxy	1	10 Jul.	25 Jul.	-	3^c^	-
81	Foxy	4	12 Jul.	04 Agu.	-	3^c^	3
240XR-6	No	3	22 Jul.	19 Agu.	14.50 ^b−h^	3^c^	3
36XL-95	No	4	29 Jul.	15 Sep.	14.14 ^c−j^	3^c^	3
37XD-25	No	4	22 Jul.	26 Agu.	12.50 ^h−o^	3^c^	3
37XM-8	No	4	24 Jul.	29 Agu.	14.71 ^b−g^	3^c^	3
37XT-163	No	4	05 Agu.	15 Sep.	-	5^b^	3
40XB-99	No	6	14 Jul.	04 Agu.	14.83 ^b−f^	3^c^	3
40XD-160	No	1	15 Agu.	15 Sep.	14.28 ^b−h^	3^c^	3
40XD-17	No	1	11 Agu.	22 Agu.	12.44 ^h−o^	3^c^	3
40XD-85*	No	1	15 Agu.	30 Sep.	13.25 ^e−m^	3^c^	3
40XD-91	No	1	13 Agu.	30 Sep.	14.25 ^c−ı^	3^c^	3
40XT-20	No	5	11 Agu.	29 Agu.	11.00 ^n−p^	3^c^	3
41XGG-1	No	2	08 Agu.	08 Sep.	-	3^c^	3
41XGG-3	No	3	28 Jul.	25 Agu.	12.05 ^j−p^	3^c^	3
41XGG-6*	No	5	05 Agu.	08 Sep.	15.50 ^a−d^	3^c^	3
41XM-10	No	5	05 Agu.	15 Sep.	13.28 ^e−m^	5^b^	3
41XT-25	No	5	25 Jul.	22 Agu.	15.65 ^a−d^	3^c^	3
41XT-9*	No	1	15 Agu.	30 Sep.	11.68 ^l−p^	3^c^	3
43XB-103	No	4	22 Jul.	25 Agu.	10.76 ^o−p^	3^c^	3
43XB-119	Light Muscat	1	14 Jul.	04 Agu.	15.16 ^b−e^	3^c^	3
43XB-41	No	4	15 Agu.	30 Sep.	12.18 ^ı−p^	3^c^	3
43XB-42	No	1	15 Agu.	08 Sep.	10.66 ^o−p^	3^c^	3
43XB-84*	No	4	25 Jul.	19 Agu.	14.16 ^c−ı^	3^c^	3
44XD-8*	Herbacous	1	09 Jul.	08 Agu.	12.71 ^g−o^	3^c^	3
44XD-12	No	1	10 Jul.	28 Jul.	10.14 ^p^	3^c^	3
44XD-25	No	1	09 Jul.	04 Agu.	13.75 ^d−l^	3^c^	3
44XD-31*	Herbacous	1	09 Jul.	04 Agu.	11.71 ^l−p^	3^c^	3
44XD-40	No	1	09 Jul.	04 Agu.	12.71 ^g−o^	3^c^	3
44XT-26	No	1	09 Jul.	04 Agu.	17.28 ^a^	3^c^	3
46XL-8*	No	1	08 Agu.	29 Agu.	14.3 ^b−h^	3^c^	3
46XM-3	No	5	15 Agu.	30 Sep.	12.00 ^k−p^	3^c^	3
46XM-4	No	1	29 Jul.	22 Agu.	11.75 ^l−p^	3^c^	3
46XT-10*	No	1	05 Agu.	08 Sep.	12.00 ^k−p^	3^c^	3
46XT-2	No	4	15 Agu.	08 Sep.	13.71 ^d−l^	3^c^	3
7XB-60	Muscat	1	11 Agu.	25 Agu.	15.15 ^b−e^	3^c^	3
7XB-79	Light Muscat	1	20 Jul.	08 Agu.	11.75 l-p	7a	3
7XC-137	No	4	19 Jul.	25 Agu.	11.35 ^m−p^	3^c^	3
7XD-258	Muscat	1	18 Agu.	30 Sep.	-	3^c^	3
7XM-98*	No	5	05 Agu.	30 Sep.	16.37 ^a−b^	5^b^	3
ALFXM-3	No	6	08 Agu.	29 Agu.	13.70 ^d−l^	3^c^	3
ASXM-5	No	1	25 Jul.	25 Agu.	12.05 ^j−p^	3^c^	3
EXSS-62*	Light Muscat	4	28 Jul.	29 Agu.	12.55 ^h−o^	3^c^	3
EXT-33	No	4	08 Agu.	08 Sep.	11.28 ^m−p^	3^c^	3
EXT-37	No	4	11 Agu.	30 Sep.	14.93 ^b−f^	3^c^	3
KPXAK-103*	Tannin	6	16 Agu.	15 Sep.	13.28 ^e−m^	3^c^	3
KPXAK-109*	No	6	08 Agu.	08 Sep.	11.71 ^l−p^	3^c^	3
KPXAK-21*	No	4	16 Agu.	15 Sep.	14.28 ^b−h^	3^c^	3
KPXAK-26*	No	5	17 Agu.	15 Sep.	16.16 ^a−c^	3^c^	3
TİXBARIŞ-3	No	1	10 Jul.	25 Jul.	12.91 ^f−n^	3^c^	3
KYXYİ	Foxy	4	10 Jul.	04 Agu.	14.08 ^c−k^	3^c^	3
TSXBARIŞ-31	No	1	17 Jul.	04 Agu.	15.07 ^b−e^	3^c^	3
YİXTM-3	Muscat	1	15 Jul.	08 Agu.	12.42 ^h−o^	3^c^	3
Alphonse Lavallée*	No	6	06 Agu.	04 Sep.	15.00 ^b−f^	3^c^	3
Crimson Seedless	No	3	23 Agu.	30 Sep.	15.06 ^b−e^	3^c^	3
Kyoho (4n)*	Foxy	6	06 Agu.	08 Sep.	13.16 ^e−m^	3^c^	3
Red Globe*	No	2	01 Agu.	30 Sep.	15.06 ^b−e^	3^c^	3
Superior Seedless	Unique	1	09 Jul.	25 Jul.	15.58 ^a−d^	3^c^	3
Tekirdağ Çek.	No	4	16 Agu.	04 Sep.	13.20 ^e−m^	3^c^	3
Trakya İlkeren*	No	6	07 Jul.	28 Jul.	15.71 ^a−d^	3^c^	3
*LSD (0.01)*					*2.09*	*1.34*	*n.s.*

* Seeded genotypes/reference varieties

Superscript letters indicate that there are significant differences at least at the *p* < 0.05 level in the relevant quality characteristics among hybrid genotypes/reference varieties

**Table 11:** Some phenolic compound values of some prominent hybrid genotypes and reference varieties

Variety / Hybrid	Berry Colour	Seed status	Total Phenolic Compounds (mg Gallic Acid Equivalent/Kg)	Total Anthocyanin (mg malvidin-3-glycoside/g)	TEAC DPPH (µM Trolox/g)	TEAC ABTS (µM Trolox/g )
12KY-YI (3 N)	Black	Seedless	1665.4^gh^	74.3^f^	0.98^hi^	32.26^b−e^
240XR-6	Red	Seedless	866.8^l^	72.4^f^	1.61^e−g^	12.00^h^
40XB-99	Black	Seedless	2222.3^d^	49.4^g^	1.81^c−f^	36.01^a−d^
40XD-17	Green yellow	Seedless	1880.5^e^	-	1.16^g−i^	50.60^a^
41XGG-3	Red	Seedless	918.3^l^	75.1^ef^	2.08^b−e^	20.17^e−h^
41XT-25	Dark red violet	Seedless	1456.9^ij^	86.5^e^	2.29^bc^	13.25^h^
43XB-119	Green yellow	Seedless	867.6^l^	-	1.49^f−h^	19.57^e−h^
44XT-26	Green yellow	Seedless	321.8^m^	-	1.15^g−i^	16.11^f−h^
46XL-8	Green yellow	Seeded	1762.4f^g^	-	2.16^b−d^	48.01^a^
7XB-60	Green yellow	Seedless	1431.2^j^	-	1.67^d−g^	44.48^ab^
ALFXM-3	Black	Seedless	2840.5^a^	1011.7^a^	2.06^b−e^	30.06^c−f^
EXSS-62	Grey	Seeded	2385.9^c^	163.4^b^	2.07^b−e^	27.30^d−g^
TSXBarış-31	Black	Seedless	1565.5^hi^	-	1.24^g−i^	45.73^ab^
Superior Seedless	Green yellow	Seedless	1073.3^k^	-	0.74^i^	12.29^h^
T.ilkeren	Black	Seeded	2647.3^b^	141.9^c^	2.74^a^	37.44^a−c^
Tekirdağ Seedless	Red	Seedless	1815.3^ef^	105.8^d^	1.20^g−i^	36.07^a−d^
Alphonse Lavallée	Black	Seeded	2804.1^a^	38.9^g^	2.56^ab^	38.32^a−c^
*LSD 0.01*			*109.31*	*16.93*	*0.53*	*15.11*

Superscript letters indicate that there are significant differences at least at the *p* < 0.05 level in the relevant quality characteristics among hybrid genotypes/reference varieties

### Characteristics of the clusters

According to the evaluation based on cluster weight, Red Globe (968.33 g), one of the standard varieties, was statistically the grape variety with the highest cluster weight. This was followed by the hybrid genotypes 40XD-17 (858.33), 37XT-163 (822.00), 43XB-84 (781.67), and 7XM-98 (723.33). The lowest cluster weight was obtained for genotype 9 among the polyploid hybrids (Table 7 and Additional Figure 1).

Cluster density is an important quality parameter in table grape breeding. A dense cluster structure is generally undesirable. Findings regarding cluster density are presented in Table 7.

When evaluated according to the OIV scale, four genotypes (44XD-25, 46XT-10, 36XL-95, and 40XB-99) were classified as dense clusters; this level of density is not desirable for table grapes. One genotype was classified as very loose-loose (46XT-2) on the OIV scale.

According to the number of berries per cluster, the hybrids with the highest number of berries were 43XB-103 (410.33), 44XD-12 (258.67), 37XT-163 (245.00), 7XD-258 (238.67), and 7XC-137 (232.67), respectively. The hybrid and reference varieties with the lowest number of berries were followed by the combinations with values of 9(3n) (32.67), Kyoho (35.00), TİXBARIŞ-3 (61.00), Alphonse Lavallée (75.33), and EXSS-62 (75.67), respectively (Table 7).

When the Berry Splitting Resistance data were examined, the combination with the highest splitting resistance was 44XD-8, with a value of 6339.87. The combination with the lowest splitting resistance was 7XC-137, with a value of 345.13 (Table 7).

When Peduncle Detachment Resistance was examined, the highest value was found in the reference variety Crimson Seedless (816.40). Following in the ranking was the hybrid combination 44XD-8 (600.60). The lowest Peduncle Detachment Resistance was found to be that of the hybrid combination 44XD-12 (6.40) (Table 7).

### Berry and quality characteristics

The weights of 10 randomly selected berries from harvested clusters were determined in mm using a precision balance. The berry weight data are given in Table 8 and Additional Figure 2.

Among the hybrids examined, the highest berry weights were 41XGG-6 (8.66), EXSS-62 (7.50), and 44XD-8 (7.19). The hybrids with the lowest berry weights were 43XB-103 (1.42), 43XB-41 (1.67), and 81 (2.09).

Data on berry width are also presented in Table 8. Among the hybrids examined, the three with the highest berry widths were 240XR-6 (25.40), 41XGG-6 (25.10), and 40XD-85 (22.03). The hybrids with the smallest berry width were KPXAK-21 (12.43), 43XB-103 (13.07), and 43XB-41 (13.80).

When berry length was examined, the hybrid genotypes with the longest berries were 44XD-8 (29.60), KPXAK-26 (29.20), and KPXAK-103 (28.90), respectively. The hybrid genotypes with the lowest berry length were 43XB-119 (14.20), 43XB-103 (14.90), and 81 (15.40), of which genotype 81 has triploid characteristics (Table 8 and Additional Figure 2).

The berries on the harvested bunches were scored for colour uniformity. Analysis of the berry colour uniformity data revealed that 10 hybrid genotypes lacked uniform colour structure, whereas 55 genotypes exhibited uniform colour structure (Table 8 and Additional Figure 2).

When the TSS (%) values were examined, the highest value was obtained from the KPXAK-21 hybrid genotype (25.50). Following this, the highest values were obtained from the 4(3n) (24.80) and KPXAK-26 (23.90) hybrid genotypes, respectively. The lowest TSS (%) values were found in the 44XD-8 (13.30), EXSS-62 (13.70), and 44XT-26 (13.90) hybrid genotypes (Table 8 and Additional Figure 2).

When the total acidity (g/L) values were examined, the highest titratable acidity was obtained for TSXBARIŞ-31 (12). The lowest acidity values were obtained by the 44XD-25 (8.50) and 44XD-12 (8.07) hybrid genotypes, which had similar values (Table 8 and Additional Figure 2).

In terms of maturity index values, the hybrid with the lowest maturity index was TSXBARIŞ-31, with a value of 9.72. The hybrid with the highest maturity index was KPXAK-21 with a value of 53.13 (Table 8 and Additional Figure 2).

In terms of seed number, the highest seed number was observed in the hybrid genotypes 44XD-40 (4.1) and 40XD-160 (3.9). No seeds were detected in six triploid genotypes and the hybrid genotypes 46XT-2 and KYXYI (Table 9).

According to the single-seed dry weight values, genotypes with a dry seed weight below 20 mg were considered seedless. Of the 55 hybrids, 41 were considered seedless, while 14 were classified as seeded grapes. A total of 7 reference varieties were selected for comparison of hybrid genotypes; 4 were seedless, and 2 were seeded. When the single seed dry weight values were examined, the genotype with the highest dry weight was found to be the Alphonse Lavallée (44.26) reference variety. This was followed by Kyoho (43.92) and 7XM-98 (41.87), respectively. The genotypes with the lowest dry weight were TSXBARIŞ-31 (0 mg), 46XT-2 (0 mg), KYXYİ (0 mg), and six polyploid genotypes (Table 9; Fig. 3).

When evaluating the results obtained from the grapes whose color values were analyzed, the lowest L* (brightness) value was obtained from the reference variety Trakya İlkeren (28.29), which has a dark berry color, while the highest value was obtained from the reference variety Superior Seedless (47.78), which has a light color. Regarding the a* (red/green coordinate) value, the lowest value was obtained from the reference variety Superior Seedless (-4.32), which has a light color, and the highest value was obtained from the reference variety Crimson Seedless (4.94), which has a red color. For the b* values, which give the yellow/blue coordinate, the lowest value was observed in the 43XB-41 hybrid genotype (-4.83), and the highest was observed in the reference variety Superior Seedless (14.02). When the chroma (brightness) values were examined, the lowest value was obtained from the triploid 14 hybrid genotype (1.62), and the highest value was obtained from the light-colored reference variety, Superior Seedless (14.61). For the hue values, the lowest value was obtained from the 41XGG-1 (50.05) hybrid genotype, and the highest value was obtained from the 41XGG-3 (326.50) hybrid genotype (Table 9 and Additional Figure 2).

Based on OIV 236 scale values, specific taste data revealed that 8 genotypes had a foxy aroma, 3 had a herbaceous aroma, 5 had a muscat aroma, and 47 had no specific taste.

Additionally, one genotype had a tannic taste, and another had a unique aroma (Fig. 4).

Regarding veraison dates, the earliest veraison was on July 9th, and the latest was on August 23rd. For harvest dates, the earliest was July 25th and the latest was September 30th (Additional Figure 4).

**Fig. 5:**
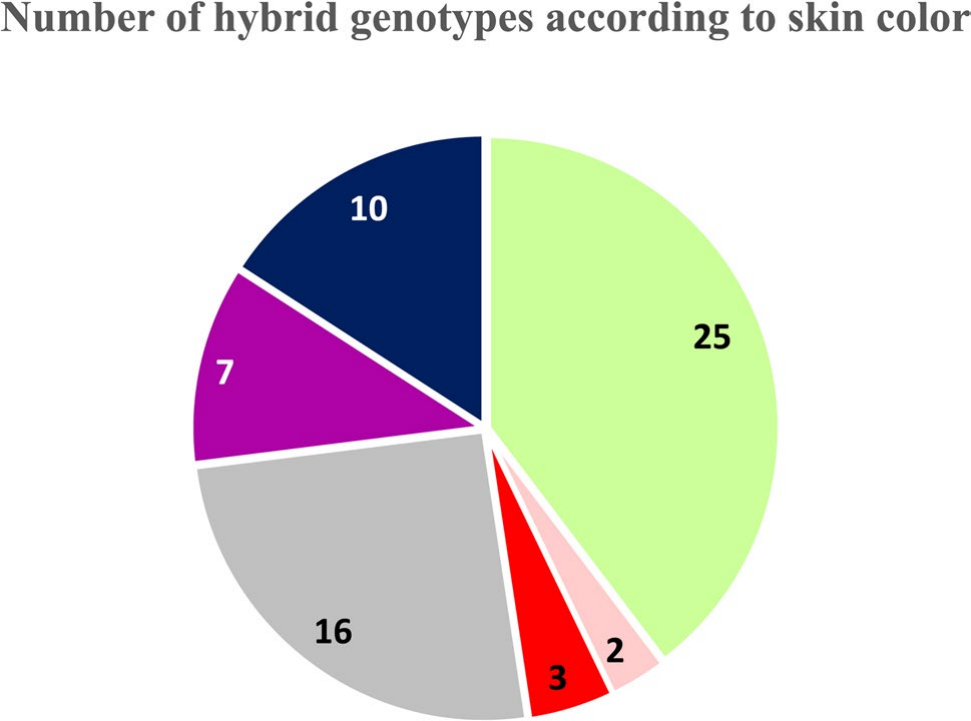
Number of hybrid genotypes/varieties according to skin colour, shown in a pie chart

According to the berry skin colour data based on the OIV 225 scale, 25 genotypes were determined to have a skin colour scale values of 1 (green yellow), 2 genotypes of 2 (rose), 3 genotypes of 3 (red), 16 genotypes of 4 (grey), 7 genotypes of 5 (dark red violet), and 10 genotypes of 6 (blue black) (Table 10; Fig. 5).

**Fig. 6:**
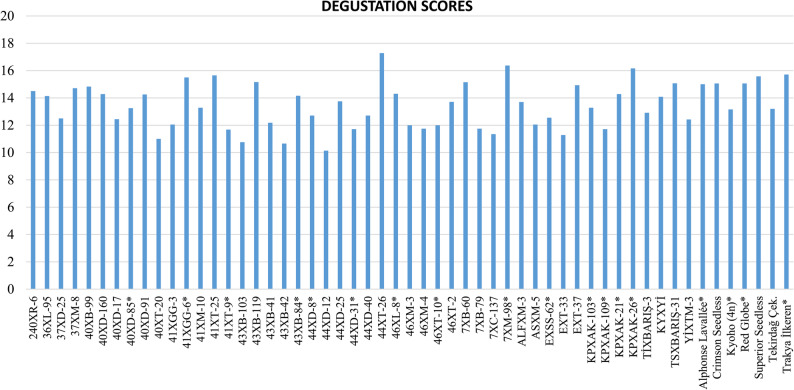
Degustation test scores of hybrids and reference varieties. (Hybrid genotypes scoring 14 and above were particularly evaluated as potential variety candidates)

According to the degustation(sensory) test results, the highest score was obtained by the seedless hybrid genotype 44XT-26 (17.28). This genotype was followed by 7XM-98 (16.37) and KPXAK-26 (16.16), respectively. The lowest sensory test results were observed in the hybrids 44XD-12 (10.14), 43XB-42 (10.66), and 43XB-103 (10.76) (Table 10; Fig. 6).

According to the assessment of powdery mildew disease based on OIV-456 criteria, genotype 12, a triploid genotype, was found to be significantly affected by powdery mildew with a value of 7, while other genotypes and reference varieties were found to be less affected with a value of 3. This is mainly due to the rainless and relatively low-humidity weather conditions throughout the production season.

For *Botrytis cinerea* disease, the OIV-459 criteria were considered. The 7XB-79 hybrid genotype received a value of 7, while the 7XM-98, EXT-37, and 41XM-10 hybrid genotypes received a value of 5, making them the most affected. Other genotypes received a value of 3, indicating less affected *Botrytis* (Table 10).

**Fig. 7:**
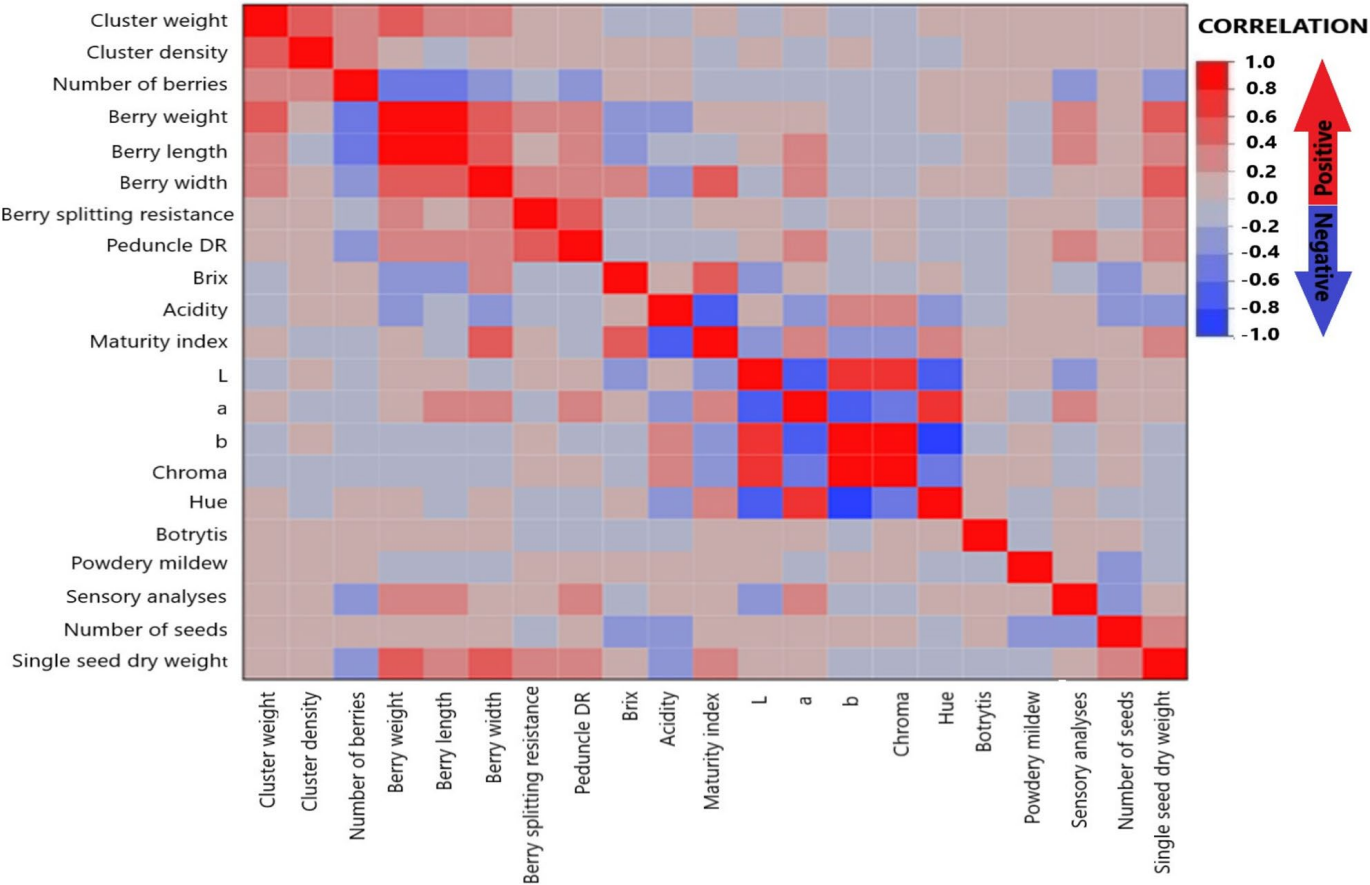
Correlation graph of important criteria used in evaluating hybrids and reference varieties

**Fig. 8:**
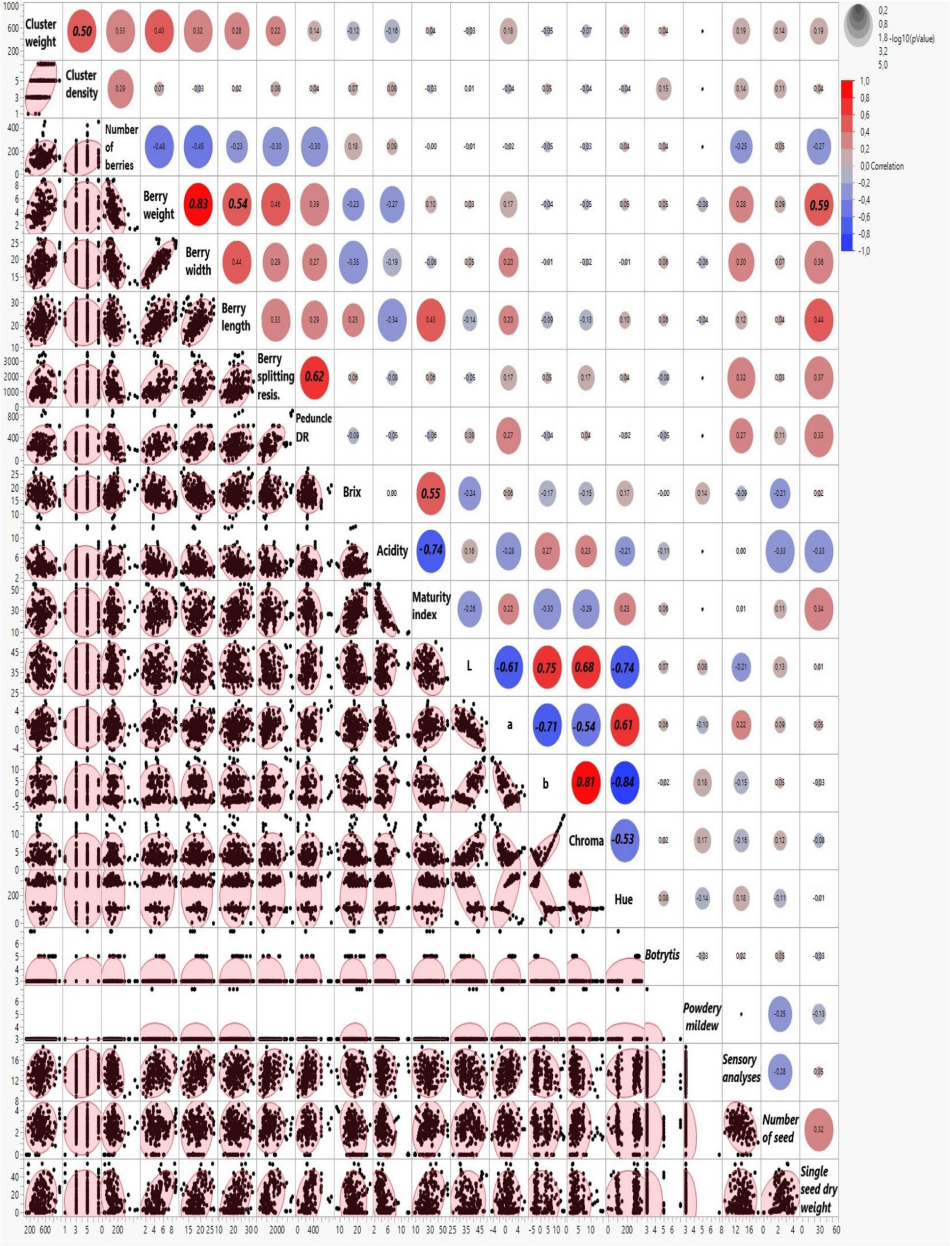
Correlation matrix of key criteria used in the evaluation of hybrid and reference varieties (with numerical values)

The correlation graph and matrix in Figs. 7 and 8 show the relationships among quality parameters. In particular, a very high positive correlation (0.83) was found between grain weight and grain size. High correlations were also observed between many color values. A very high positive correlation (0.81) was found between b* and Chroma, a high positive correlation (0.61) between Hue and a*, and a high negative correlation (-0.71) between a* and b*. A high positive correlation (0.62) was also found between berry splitting resistance and peduncle PR.

**Fig. 9:**
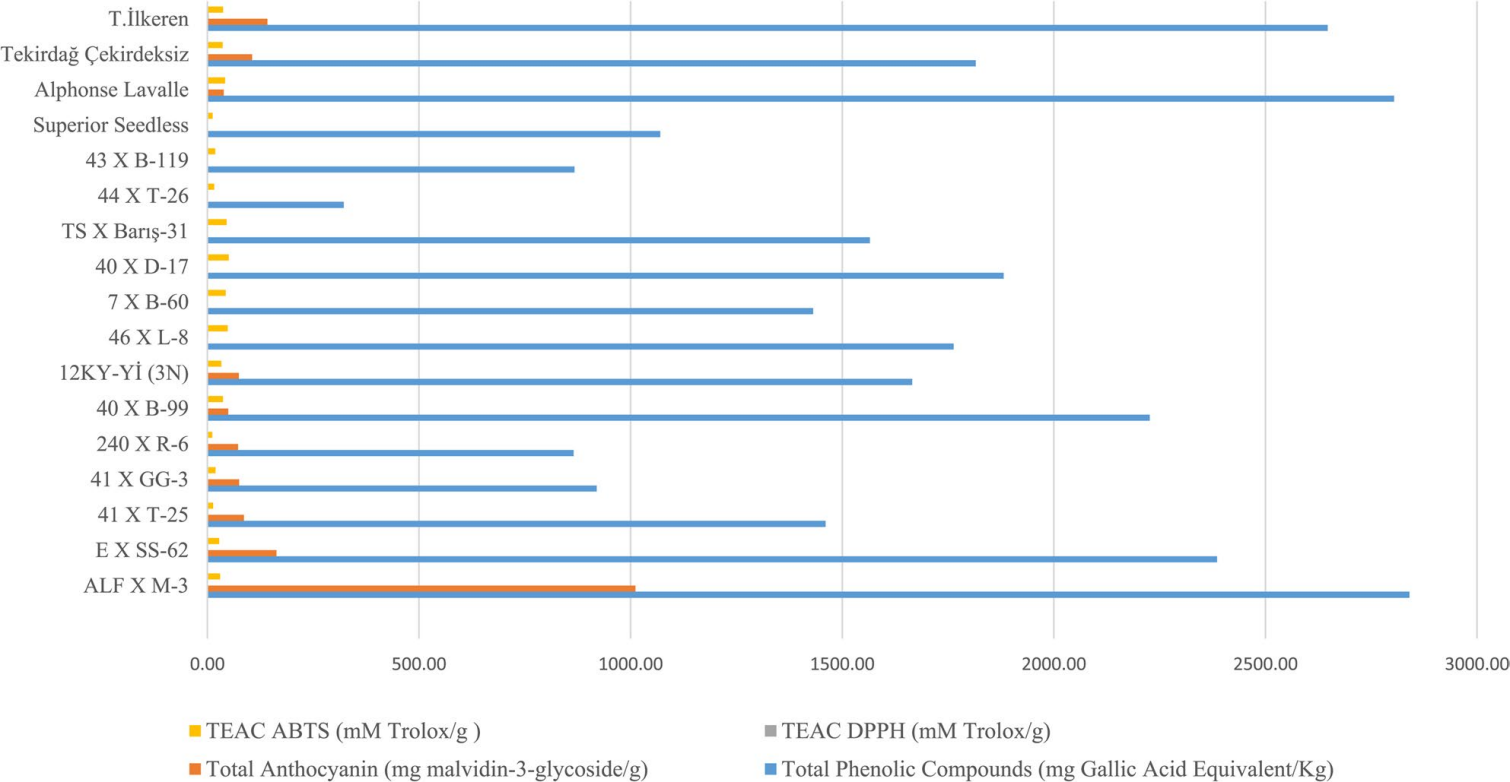
Phenolic compound values of prominent hybrid genotypes and reference varieties on a clustered bar graph

Table 11; Fig. 9 present phenolic component values of prominent hybrid genotypes and reference varieties with sufficient cluster numbers. The highest total phenolic compound content was obtained from the black-colored, seedless ALFXM-3 hybrid genotype and the reference variety Alphonse Lavallée, while the lowest value was obtained from the light red-colored, seeded 44 X T-26 hybrid genotype. The highest total anthocyanin content was obtained from the black seedless hybrid genotype ALFXM-3. In terms of total antioxidant content, using two different methods, the highest values were obtained from the seedless 40XD-17 and the seeded 46XL-8 genotypes, as well as the reference variety Trakya Ilkeren.

## Discussion

Cross-breeding studies in grapes, as in this study, are quite long-term research studies that begin with the emasculation of female parents and continue with the analysis of the first bunches after about 5–6 years (Additional Fig. 5). The results obtained from hybrid grape genotypes resulting from hybridization breeding studies, particularly regarding cluster, berry, seed, taste, and fungal disease characteristics, were compared with similar studies. While cluster density assessments are more prominent in wine grapes, it is also an important quality criterion considered in table grapes. As in this study, breeding efforts aim to develop new hybrid genotypes that do not have very dense clusters [26].

In our study, we determined that the parents with a dense cluster structure were generally Tekirdağ Seedless, Ribol, Güz Üzümü, Alphonse Lavallée, Bozbey, and Trakya İlkeren grape varieties. It was reported that the cluster density structure of hybrid combinations varied depending on the parents. In a study by Ergönül et al. [27], the authors reported that parent selection affected cluster density in a manner similar to our results. They reported that individuals with medium cluster density were observed in most hybrid combinations, but combinations using the İrikara variety as a parent formed denser clusters. They also reported that individuals belonging to Hönüsü x Perlette combinations formed sparser cluster structures.

Another study with similar results to ours was conducted by Sharma et al. [28] on a Riesling × Cabernet Sauvignon combination. The researchers reported that the cluster architecture changed depending on the varieties used as parents. They observed that hybrids using Riesling as a parent developed smaller, denser, and fewer clusters with smaller berries; while hybrids using Cabernet Sauvignon as a parent developed larger, looser, and more numerous clusters with larger berries. When they determined the number of berries in the cluster using imaging techniques, they found that Riesling produced fewer berries (37.2-34.37), whereas Cabernet Sauvignon produced more (58.56–55.66). Significant differences were observed between the parents in terms of cluster density. Riesling showed higher cluster density, while Cabernet Sauvignon exhibited lower cluster density. In line with this literature, our study also determined significant differences in cluster density and berry number among hybrid genotypes. It was determined that these characteristics originated primarily from the parents’ traits.

Atak et al. [21] examined the important quality parameters of 23 grape hybrids crossbred by two different institutes in two different harvest years. In their study on berry width (mm), they reported values of 15.4–22.3 mm in 2008 and 15.2–25.0 mm in 2009, showing a wide range of values. A similar study was conducted by Kamiloğlu et al. [29] in Hatay with some grape varieties and variety candidates. The researchers reported that berry width ranged from 15.19 to 21.10 mm and berry length ranged from 18.17 to 22.86 mm. In our study, although the berry width values of the hybrid genotypes were close to those reported by the researchers (12.43–25.4 mm), they showed a wider range.

Wang et al. [30], in their study examining the quality parameters of 17 different table grape varieties, determined that soluble solids content (SSC) ranged from 15.71% to 21.17%. The data for the new variety Ruby Seedless (21.17%) were higher than those of the other varieties.

Maante-Kuljus et al. [31], in an examination of the quality characteristics of 3 hybrid grape varieties under 2 different growing conditions over 3 years, reported that SSC values ranged from 13.8% to 25.4%. In our study, however, the SSC values obtained from hybrid genotypes were found to have a wider range (8.9%-25.5%). This may be due to differences in environmental conditions, the parent varieties used, and harvest dates. Indeed, Erez et al. [32], in their study they determined similar SSC ranges across 20 different grape varieties grown in Siirt province and attributed this to factors such as vineyard maintenance, differences in rainfall amounts, temperature, and other ecological factors between years.

In our study, acidity values obtained from hybrid genotypes also showed a wide range, similar to those of SSC. Based on these criteria, the maturity index values obtained from hybrid genotypes also had a wide range. Cangi and Altun [33] reported that maturity index values ranged from 34.6 to 47.3 across different grape varieties in their study. Bozkurt et al. [34] reported that maturity index values ranged from 22.4 to 33.3 in their study with 15 grape varieties over 2 years. It is reported that maturity index values should be evaluated based on the variety’s harvest time and evaluation method, and that a value of around 20 is suitable for harvesting, especially for early varieties [35].

In table grape breeding studies, intensive efforts are made to obtain early and late varieties that are harvested outside the normal season. These varieties are particularly important for achieving higher prices and generating more income. Therefore, one aim of many breeding studies is to obtain varieties that are harvested early or late. Balic et al. [36] and Guo et al. [37] in their breeding studies aimed at developing early varieties, developed hybrid genotypes that could be harvested early and reported that their market share was quite high. In our study, since early- and late-ripening hybrid genotypes are important, hybridizations were carried out by selecting suitable parents. Consequently, a difference of more than 2 months was observed between the earliest and latest harvested hybrid genotypes.

Kamiloğlu [37], in his study to determine the berry quality characteristics of some early table grapes, found berry detachment resistance to range from 576.83 to 332.92 g. In the same study, they determined the berry splitting resistance to be between 2672.92 and 1007.67. In another similar study, Yıldız et al. [38] examining the quality parameters of some table grape varieties and clones, reported that berry splitting resistance ranged from 1830 g to 3090 g, and berry detachment resistance ranged from 310 g to 840 g. In our study, the berry splitting resistance data ranged from 345 to 3406.67, and the berry detachment resistance data ranged from 6.4 to 816.4. The use of different parents and a larger number of hybrid genotypes in our study contributed to the slightly wider range.

Rolle et al. [39] in a study conducted on 10 white grape varieties (2011), they examined the L, a, b, hue (H), and chroma (C) values. They reported that the L value ranged from 37.69 to 42.01, the a value from (-5.59) to (-1.91), the b value from 9.55 to 15.29, the chroma (C) value from 16.30 to 10.33, and finally, the hue (H) value from 100.09 to 113.33. Rolle et al. [40] examined the L, a, b, hue (H), and chroma (C) values in another study using standard varieties. The data for L values ranged from 28.57 to 25.68, a values from 1.24 to 5.61, b values from − 0.44 to 0.65, chroma (C) from 1.32 to 5.68, and hue (H) from (-14.42) to 4.79. In our study, when examining the L, a, and b values, the L value ranged from 28.29 to 47.77, a value from − 4.32 to 4.94, b value from (-4.83) to 14.02, chroma (C) from 1.62 to 14.68, and hue (H) from 50.05 to 326.5. The presence of both red and green shell genotypes in our study resulted in a wider, higher range of L values, ranging from 28.29 to 47.77. The observed differences in L, a, b, hue (H), and chroma (C) values are because, while all the varieties examined in Rolle et al. [40] study were red-skinned, our study included varieties with both red and green skins. Seedlessness is one of the most sought-after characteristics in table grapes, and the demand for seedless table grapes is constantly increasing. Therefore, developing new seedless varieties was among the main objectives of this breeding study. In line with this objective, 72.9% of the 59 hybrids in our study were seedless, while 27.1% had seeds. Barış and Gürnil [41] studied the inheritance of seedlessness in genotypes derived from hybridizing 22 seeded and 3 seedless (Sultani seedless, Perlette, Beauty Seedless) varieties. 1229 individuals with seeds and 1142 seedless individuals were identified, and researchers reported that the parents’ effects on seedlessness were observed. They reported that in hybridizations with Perlette, 42.8% of individuals were seedless and 57.2% were seedless.

Similarly, in our study, six of the eight hybrids where Perlette was used as a parent were seedless, and two were seeded. In conclusion, using Perlette as a parent resulted in a higher seedlessness rate than reported in the literature, and it was concluded that it is a good parent for seedless breeding studies.

Fruit shape, an important defining characteristic in breeding studies, is a crucial selection criterion for developing new varieties to meet significant market demands. In evaluating the shape of hybrid genotypes, researchers generally used the OIV 223 scale, as in our study. Based on observations and analyses, it was determined that the hybrids had different seed shapes. Zhang et al. [42] investigated the shapes of grape berries across varieties. They reported that differences in berry shape could mainly be attributed to differences in the growth rates of the fleshy cells in the longitudinal and transverse directions. They also reported that these different growth rates could result in differences in berry length and width.

Bozkurt et al. [34] in their study on different grape varieties under Erzincan conditions, reported that bunch weight ranged from 191.7 to 480 g and berry weight ranged from 2.2 to 6.9 g. Souza et al. [43] examined the phenological and morphological data of 5 hybrid vines and stated that bunch weights ranged from 175 to 278 g and berry weights ranged from 2.3 to 2.9 g. In another study, Özdemir et al. [44] reported that bunch weight values of 15 seeded and seedless grape varieties grown in Adana ranged from 149 to 478 g, and berry weight ranged from 1.50 to 7.20 g. In our study, bunch weight ranged from 253 to 858 g, and berry weight ranged from 1.42 to 8.66 g. The slight difference from similar studies may be due to the large number of hybrids studied, vineyard irrigation, and the first year of yield.

In their study on the post-harvest quality of some wine varieties, Kamiloğlu and Üstün [45] found a very low positive correlation (0.32) between the number of seeds and their dry weight. Similarly, a very low positive correlation was observed in our study. When the correlations among all the quality parameters examined in our study were evaluated, the results were similar to those reported in the literature. A positive and strong correlation (0.62) was found between the berry detachment force from the stem and the berry splitting resistance. This value was found to be quite close to the value reported by Kamiloğlu [46] (0.65). This indicates that there may be a physiological parallelism between berry splitting resistance and berry detachment force from the stem. The high positive correlation (0.55) between water-soluble dry matter and the maturity index is similar to the value (0.63) reported by the researchers.

In line with the high negative correlation (-0.74) we found between maturity index and acidity, Kamiloğlu [46] also reported a very high negative correlation (-0.94). This study confirms the tendency for the maturity index to increase with decreasing acidity during the ripening period. The high positive correlation (0.83) we found between berry weight and berry width was also observed in Kamiloğlu’s study (0.94). In general, similar positive correlations were observed in both studies. Atak et al. [18] in their study examining the important quality characteristics of 89 hybrid grape genotypes, reported that cluster-related characteristics showed strong positive relationships with one another; they also reported similar correlations among criteria related to fruit size/dimensions and seed number. They also reported that color values showed strong positive and negative correlations due to their inverse relationships. Although similar results were observed across the same quality criteria in our study, differences were observed in the numerical correlation values. Although the directions of the correlations were similar, the values in the literature were found to be higher.

This may be due to differences in materials and growing conditions. While the literature studies 6 standard grape varieties in the Hatay ecology, our study used 59 hybrid and 6 standard varieties in the Tekirdağ ecology, thereby providing greater genetic variation. It is thought that this greater genetic diversity may have led to lower correlation numbers. In addition, the fact that the harvest time in our study spanned July to September, and the evaluation of genotypes with different ripening times together, may have led to differences in these correlation values.

Researchers have reported that the levels of different phenolic compounds and anthocyanins in grape berries can vary not only by *Vitis* species but also by variety [47–49]. Furthermore, these contents can vary considerably in different parts of the fruit (skin, flesh, and seeds) [50]. In addition, consistent with our results, it has been reported that varieties or hybrids with dark-colored fruits may have higher total phenolic content than those with light-colored fruits [51].

## Conclusion

Table grape breeding studies must continue uninterrupted to develop new varieties that meet changing consumer demands. Especially in recent years, consumers have been demanding new varieties that are seedless, large-berry, have firm flesh, a unique aroma, and are more tolerant or resistant to diseases and pests. Our study was focused on these consumer demands and also conducted to increase genetic diversity in table grape breeding and contribute to the development of new varieties with superior characteristics. As a result of our study, we obtained many hybrid genotypes with diverse characteristics. In particular, hybrid genotypes with early ripening, seedless, disease-tolerant, and large berries stood out due to their high market potential. 44XD-8, 44XT-26, and 7XM-98 were the most noteworthy hybrid genotypes. The seeded KPXAK-26 and seedless 41XT-25, 41XGG-6, 7XB-60, 43XB-103, 43XB-119, and TSXBARIŞ-31 hybrid genotypes, which scored 15 or higher in sensory tests, attracted attention; further studies and more comprehensive analyses are planned to reveal the potential of these varieties. Hybrid genotypes that can compete with, or even surpass, standard varieties will have their true potential and market value fully understood if examined through more comprehensive trials across different ecological conditions. Subsequent studies, particularly focusing on storage and shelf life, will provide clearer information about their chances of success in domestic and foreign markets.

## Supplementary Information

Supplementary Material 1.

Supplementary Material 2.

Supplementary Material 3.

Supplementary Material 4.

Supplementary Material 5.

## Data Availability

The datasets used and/or analysed during the current study are available from the corresponding author on reasonable request.
