# Supplementary figures and images for: Comparison of key quality characteristics and table grape potentials of diploid and triploid hybrid genotypes obtained through cross-breeding
Source: BMC Plant Biol. 2026 Jul 24;26:1257. doi: 10.1186/s12870-026-09389-7 (PMC13401300; doi:10.1186/s12870-026-09389-7)

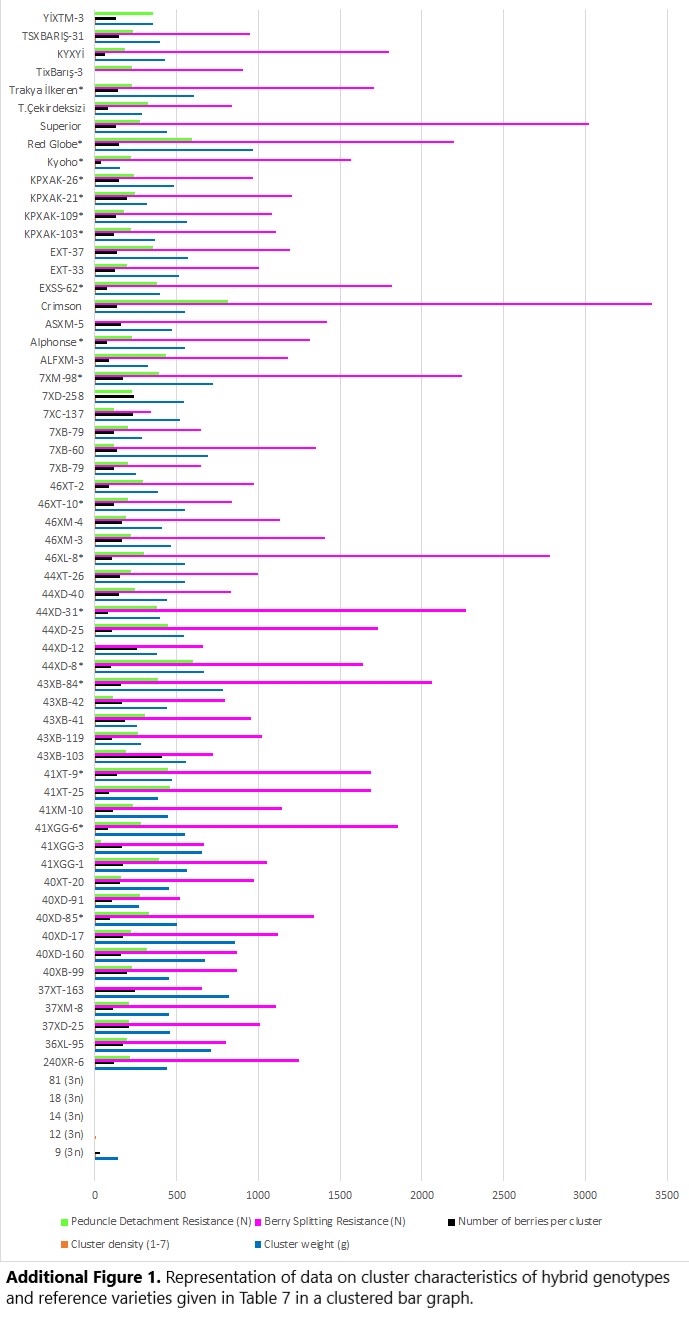

Supplement: Supplementary file 1 — Supplementary Material 1. [file 12870_2026_9389_MOESM1_ESM.jpg]

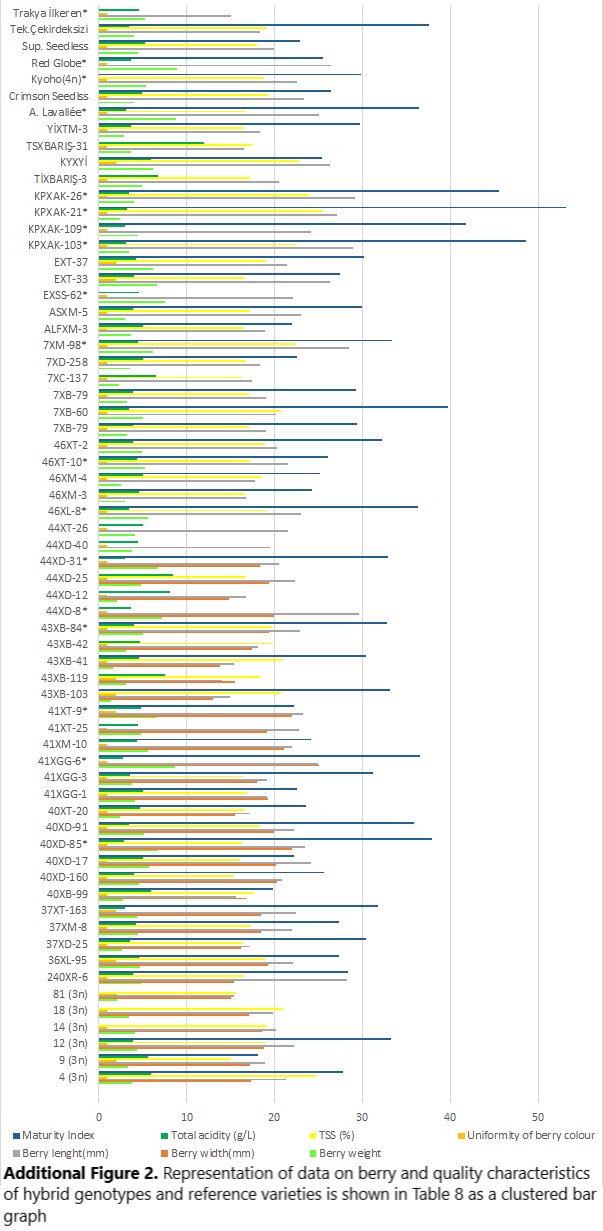

Supplement: Supplementary file 2 — Supplementary Material 2. [file 12870_2026_9389_MOESM2_ESM.jpg]

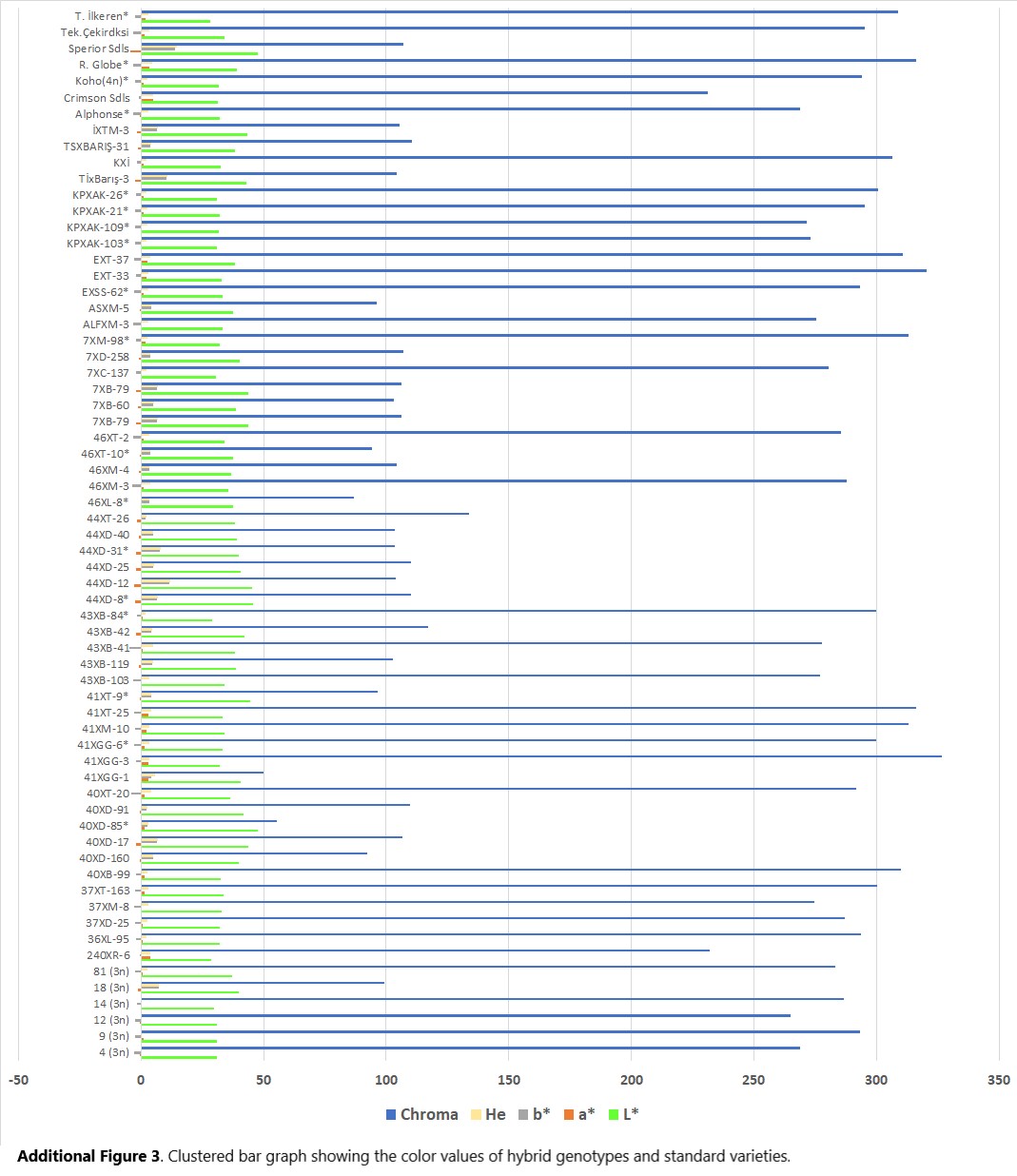

Supplement: Supplementary file 3 — Supplementary Material 3. [file 12870_2026_9389_MOESM3_ESM.jpg]

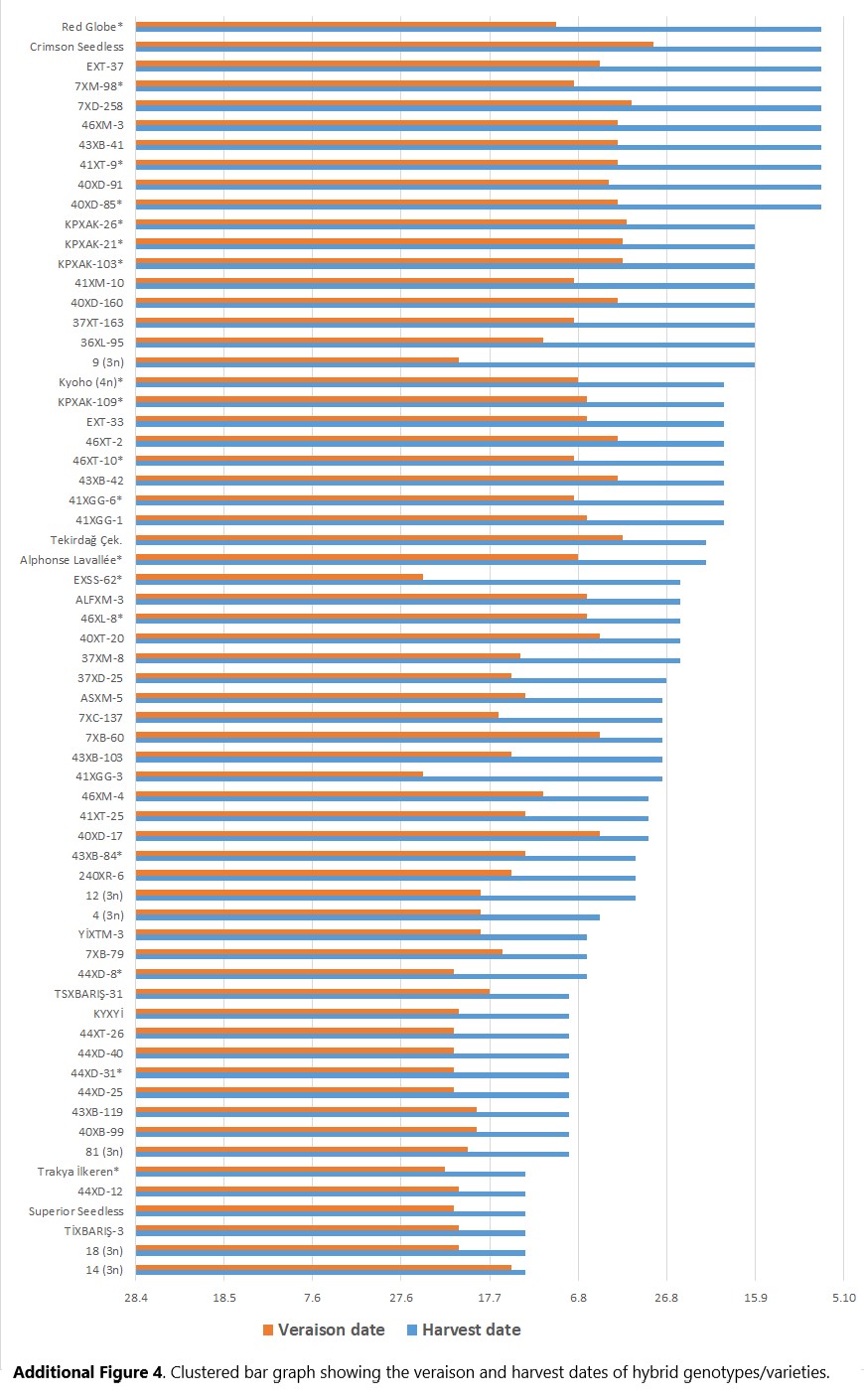

Supplement: Supplementary file 4 — Supplementary Material 4. [file 12870_2026_9389_MOESM4_ESM.jpg]

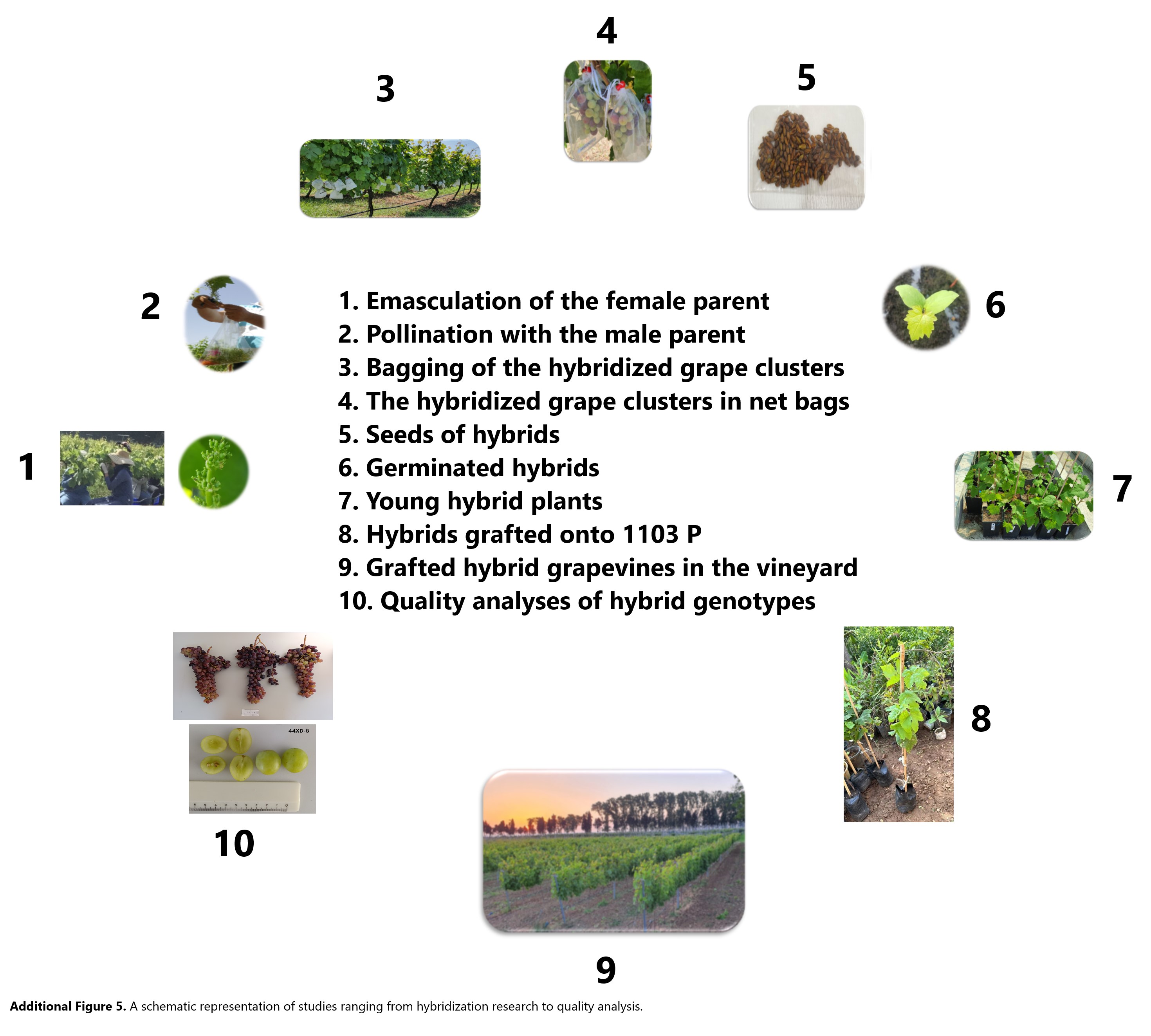

Supplement: Supplementary file 5 — Supplementary Material 5. [file 12870_2026_9389_MOESM5_ESM.jpg]
